# How to Make the Stress Relaxation Experiment for Polymers More Informative

**DOI:** 10.3390/polym15234605

**Published:** 2023-12-02

**Authors:** Anna Stankiewicz, Sławomir Juściński

**Affiliations:** 1 Department of Technology Fundamentals, Faculty of Production Engineering, University of Life Sciences in Lublin, 20-612 Lublin, Poland; 2 Department of Power Engineering and Transportation, Faculty of Production Engineering, University of Life Sciences in Lublin, 20-612 Lublin, Poland; slawomir.juscinski@up.lublin.pl

**Keywords:** viscoelasticity of polymers, linear relaxation modulus, stress relaxation test, experiment randomization, differentiable Lipchitz models

## Abstract

Different viscoelastic models and characteristics are commonly used to describe, analyze, compare and improve the mechanical properties of polymers. A time-dependent linear relaxation modulus next to frequency-domain storage and loss moduli are the basic rheological material functions of polymers. The exponential Maxwell model and the exponential stretched Kohlrausch–Williams–Watts model are, probably, the most known linear rheological models of polymers. There are different identification methods for such models, some of which are dedicated to specific models, while others are general in nature. However, the identification result, i.e., the best model, always depends on the specific experimental data on the basis of which it was determined. When the rheological stress relaxation test is performed, the data are composed of the sampling instants used in the test and on the measurements of the relaxation modulus of the real material. To build a relaxation modulus model that does not depend on sampling instants is a fundamental concern. The problem of weighted least-squares approximation of the real relaxation modulus is discussed when only the noise-corrupted time-measurements of the relaxation modulus are accessible for identification. A wide class of models, that are continuous, differentiable and Lipschitz with respect to parameters, is considered for the relaxation modulus approximation. The main results concern the models that are selected asymptotically as the number of measurements tends to infinity. It is shown that even when the true relaxation modulus description is completely unknown, the approximate optimal model parameters can be derived from the measurement data that are obtained for sampling instants that are selected randomly due to the appropriate randomization introduced whenever certain conditions regarding the adopted class of models are satisfied. It is shown that the most commonly used stress relaxation models, the Maxwell and Kohlrausch–Williams–Watts models, satisfy these conditions. Since the practical problems of the identification of relaxation modulus models are usually ill posed, Tikhonov regularization is applied to guarantee the stability of the regularized solutions. The approximate optimal model is a strongly consistent estimate of the regularized model that is optimal in the sense of the deterministic integral weighted square error. An identification algorithm leading to the best regularized model is presented. The stochastic-type convergence analysis is conducted for noise-corrupted relaxation modulus measurements, and the exponential convergence rate is proved. Numerical studies for different models of the relaxation modulus used in the polymer rheology are presented for the material described by a bimodal Gauss-like relaxation spectrum. Numerical studies have shown that if appropriate randomization is introduced in the selection of sampling instants, then optimal regularized models of the relaxation modulus being asymptotically independent of these time instants can be recovered from the stress relaxation experiment data. The robustness of the identification algorithm to measurement noises was demonstrated both by analytical and numerical analyses.

## 1. Introduction

Various rheological models have been widely adopted to describe the combined elastic and viscous properties of various polymers for a hundred years [1,2]. A time-dependent linear relaxation modulus next to frequency-domain storage and loss moduli are the basic rheological material functions of polymers. The viscoelastic behavior of polymers varies depending on the type of polymer [1,3,4,5,6]; therefore, different models have been and are still being developed. The exponential relaxation is often modeled using Maxwell models [2,3,7]. When the Debye decays show deviations from pure exponential solutions, it can be approximated by the exponential stretched Kohlrausch–Williams–Watts (KWW) model [8,9].

A model of the relaxation modulus can be recovered from the experiment data by applying an appropriate identification method. Identification consists of the selection, within the given class of models, of such a model, which ensures the best fit to the measurement results. This paper deals with the problem of the recovery of the relaxation modulus model of linear viscoelastic material from discrete-time noise-corrupted measurements that are obtained in the stress relaxation test. The viscoelastic relaxation modulus identification problem is not aimed at achieving a true description of the real relaxation modulus, but one that is a “sufficiently or optimally accurate”. A model is never a true description of the real material, as a model will always contain errors and discrepancies compared with the real rheological process behavior. Therefore, a model is always only the approximation of the true material description [10].

For model identification, three entries are necessary: the measurement data from the real material, the set of models within which the required model is sought, and the identification criterion for the best model selection [10]. When the set of models is selected based on the properties of the studied material, the model that is chosen depends on the experimental data and the identification criterion. We usually determine the parameters in a model by obtaining the “best-possible” fit to experimental data. The coefficients can be highly dependent on our way of measuring “best” [10]. A common choice of model quality measure (identification index) is the mean-square approximation error, leading to a least-squares identification problem. When the identification index is fixed, the designated model can also be highly dependent on the measurement data.

In this paper, we focused on how to identify a model that is independent of the specific time instants at which we record stress measurements in the stress relaxation experiment. Loosely speaking, the problem was whether the identification procedure will yield a relaxation modulus model that is asymptotically (when the number of measurements tends to infinity) independent of the particular sampling instants. The issue involves aspects on whether the data set (i.e., the experimental conditions) is informative enough to guarantee this convergence result.

We consider the problem of sampling-instant-independent approximation of a linear relaxation modulus of the polymer within the parametric class of models when the integral weighted square error is to be minimized and the true material description is completely unknown. We showed how the problem can be solved by introducing an appropriate randomization on the set of sampling instants at which the polymer relaxation modulus is measured. It was assumed that only the relaxation modulus measurements are accessible for identification. The problems of an optimal least-squares approximation of a relaxation modulus in the classes of usually used models are the ill-posed problems [11,12] of the best fitting of time-measured data by the finite sum of exponential functions (Maxwell models) or the exponential stretched function (KWW model). Therefore, Tikhonov regularization [11,12], combined with choosing the regularization parameter by the guarantee model approximation rule, was used to stabilize the solution of the problem. A simple identification algorithm providing the strongly consistent estimate of the optimal model was given. The stochastic-type convergence analysis was performed, and the rate of convergence was discussed for the case when the measurements are corrupted by additive noises. The idea of measurement-point-independent identification is inspired by the fundamental Ljung paper [13] and the paper [14] concerning, respectively, dynamic and static zero-memory systems’ optimal identification tasks. The results of the simulation experiments for three-, five- and seven-parameter Maxwell models and the KWW model are presented for the polymer material described by a bimodal Gauss-like relaxation spectrum, which is often used to describe the rheological properties of various polymers [15], e.g., poly(methyl methacrylate) [16], polyacrylamide gels [17] and polymers used in food technology [18,19,20]. Both asymptotic properties and robustness on noise measurements were examined.

In Appendix A, the proofs and derivations of some mathematical formulas are given, to increase the clarity of the article. Some tables related to numerical studies are moved to Appendix B.

## 2. Materials and Methods

In this section, the assumptions concerning the viscoelastic material modeled by the Boltzmann constitutive integral equation and parametric classes of models describing the linear relaxation modulus are given and discussed. The problem of the optimal approximation of the real completely unknown relaxation modulus in the assumed class of models is formulated, which by minimizing the integral weighted square error results in the optimal model that is independent of the particular measurement time instants. Next, the concept of the relaxation experiment randomization is introduced so that the successive sampling instants are selected randomly and independently with the same probability distribution. Assumptions regarding this probability distribution and measurement noises are introduced and justified. Finally, the empirical square identification index is introduced, and the optimal identification task is stated along with the respective regularization.

### 2.1. Material

We consider a linear viscoelastic material that is subjected to small deformations for which the uniaxial, nonaging and izotropic stress–strain equation is represented by a Boltzmann superposition integral [1]:

(1) $$\sigma \left(t\right)=\int_{-\infty }^{t}G\left(t-\tau \right)\dot{\varepsilon }\left(\tau \right)d\tau ,$$

where σ(t) and ε(t) denotes, respectively, the stress and strain at the time t, and G(t) is the linear (Boltzmann) relaxation modulus.

The modulus G(t) is the stress, which is induced in the viscoelastic material described by Equation (1) when the unit step strain ε(t) is imposed. By assumption, the exact mathematical description of the relaxation modulus G(t) is completely unknown. The value of G(t) can be, however, measured with a certain accuracy for any given value of the time t∈T, where $T=\left[t_{0},T\right]$, the initial time $t_{0}\geq 0$ and 0<T<∞ or $T={ℛ}_{+}$; here, ${ℛ}_{+}=[0,\infty )$.

### 2.2. Models

Throughout, we will be concerned with the case when the relaxation modulus model is to be selected within a certain parametric class of models, defined by the admissible set of parameters $G\subset {ℛ}^{K}$ (finite-dimensional parameterization) and the mapping $G_{M}:T\times G\to {ℛ}_{+}$. Thus, the relaxation modulus model is described by

(2) $$G_{M}\left(t\right)=G_{M}\left(t,g\right),$$

where the subscript ‘M’ means the model.

It is not assumed that the real relaxation modulus G(t) is represented in the chosen class of models.

### 2.3. Assumptions

We make the following assumptions:

**Assumption** **1.** *The real relaxation modulus G(t) is bounded on T, i.e., $\underset{t\in T}{sup}\;G\left(t\right)\leq M<\infty$.*

**Assumption** **2.** *The set of admissible model parameters G is compact subset of ${ℛ}^{K}$.*

**Assumption** **3.** *For any t∈T, the function $G_{M}\left(t,g\right)$ is continuous and differentiable with respect to g and so that $G_{M}\left(t,0_{K}\right)=0$, where $0_{K}$ is K-dimensional zero vector in ${ℛ}^{K}$.*

**Assumption** **4.** *$\underset{t\in T,g\in G}{sup}{\left\|\nabla_{g}G_{M}\left(t,g\right)\right\|}_{2}\leq M_{1}<\infty$, where $\nabla_{g}G_{M}\left(t,g\right)$ denotes the gradient of the function $G_{M}\left(t,g\right)$ with respect to the vector g, where ${\left\|\cdot \right\|}_{2}$ is the Euclidean norm in the space ${ℛ}^{K}$.*

Assumption 1 is natural in the context of approximation of the relaxation modulus. The parameters of known rheological models most often have a physical interpretation, they are non-negative and bounded, and therefore, Assumption 2 follows. Assumptions 3 and 4 do not apply to the material, but to its model, which to a certain extent can be chosen arbitrarily and guarantee that model $G_{M}\left(t,g\right)$ is a Lipschitz function with respect to the parameter vector g for any t∈T and should not be a restriction. The parameter vector g varies over the compact set G. Thus, for any g∈G, we have ${\left\|g\right\|}_{2}\leq M_{2}<\infty$, and Assumptions 3 and 4 yield

(3) $$\underset{t\in T,g\in G}{\sup}\left|G_{M}\left(t,g\right)\right|\leq M_{1}M_{2}=M_{3}<\infty ,$$

i.e., Assumptions 2–4 introduce the same saturation on the relaxation modulus model. The above and Assumption 1 immediately result in the estimate

(4) $$\underset{t\in T,g\in G}{\sup}\left|G\left(t\right)-G_{M}\left(t,g\right)\right|\leq M+M_{3}<\infty ,$$

which is identical with Assumption A4 in [14]. Other assumptions from [14] are also satisfied when the above conditions hold. A detailed analysis of the above assumptions for the most frequently used rheological models will be carried out below.

### 2.4. Problem of the Optimal Relaxation Modulus Approximation

The following relaxation modulus approximation problem is considered. Determine the model within the class of models defined by Equation (2) that minimizes the global approximation error of the form

(5) $$Q\left(g\right)=\int_{T}{\left[G\left(t\right)-G_{M}\left(t,g\right)\right]}^{2}\rho \left(t\right)dt,$$

where a chosen weighting function $0\leq \rho \left(t\right)\leq M_{0}<\infty$ is a density on T, i.e., $\int_{T}\rho \left(t\right)dt=1$.

Since ρ(t) is an absolutely integrable function, in view of (4), the product of a bounded function ${\left[G\left(t\right)-G_{M}\left(t,g\right)\right]}^{2}$ and ρ(t) is absolutely integrable too, regardless of the bounded or unbounded domain T. Thus, the integral (5) is well defined for any g∈G.

The problem of the relaxation modulus G(t) optimal approximation within the class of models described by (2) consists of determining the parameter $g^{*}$ that minimizes the index Q(g) over the set of admissible parameters G, i.e., it takes the form

(6) $$\underset{g\in G}{\min}\;\;Q\left(g\right)=Q\left(g^{*}\right).$$

Due to Assumption 3, the index Q(g) (5) is a continuous function of the vector g, and thus, by the Weierstrass theorem concerning the extreme of continuous function on the compact set (Assumption 2) [21], the existence of the solution to the optimization problem (6) is immediately concluded. The optimal parameter $g^{*}$ does not depend on the particular time instants; however, it obviously depends on the time domain T.

### 2.5. Relaxation Modulus Measurements

Let $T_{1},\ldots ,T_{N}$ be independent random variables with a common probability density function ρ(t), whose support is T. Let $G_{i}=G\left(T_{i}\right)$ be the corresponding relaxation modulus, i=1,…,N, and let ${\bar{G}}_{i}$ denote their measurements obtained in a certain stress relaxation test [1,2,17]. We will assume that the measurements of the relaxation modulus are corrupted by additive noise $Z_{i}$, i.e., ${\bar{G}}_{i}=G_{i}+Z_{i}$.

We assume additionally that:

**Assumption** **5.** *The measurement noise $\left\{Z_{i}\right\}$ is a time-independent, i.e., independent of the variables $\left\{T_{i}\right\}$, sequence of independent identically distributed (i.i.d.) random variables with zero mean $E\left[Z_{i}\right]=0$ and a common finite variance $E\left[Z_{i}^{2}\right]=\sigma^{2}<\infty$.*

**Assumption** **6.** *The measurement noises $Z_{i}$ are bounded, i.e., $\left|Z_{i}\right|\leq \delta <\infty$ for i=1,…,N.*

Obviously, from Assumption 5, it follows that for i=1,…,N, the expected value

(7) $$E{\left[G\left(T_{i}\right)+Z_{i}-G_{M}\left(T_{i},g\right)\right]}^{2}=Q\left(g\right)+\sigma^{2}.$$

### 2.6. Identification Task

For practical reasons, integral index Q(g) (5) can be replaced by the finite mean sum of the model square errors, i.e., by the index

(8) $$Q_{N}\left(g\right)=\frac{1}{N}\sum_{i=1}^{N}{\left[{\bar{G}}_{i}-G_{M}\left(T_{i},g\right)\right]}^{2},$$

being the familiar mean-squares criterion for relaxation modulus model (2); the lower index is the number of measurements. The empirical index $Q_{N}\left(g\right)$ is obtained by the replacement of the integral in Q(g) (5) with the finite mean sum of squares.

The problems of determining models used in polymer rheology based on measurement data are usually ill posed in the Hadamard sense [11,12], i.e., the solution to a direct minimization task

(9) $$\;\underset{g\in G}{\min}\;\;Q_{N}\left(g\right)$$

can be not unique, and small changes in measured relaxation modulus can lead to arbitrarily large changes in the determined model. The stable approximate solutions can be found by minimizing the Tikhonov functional formed by the linear combination

(10) $$L_{N}\left(g,\lambda \right)=Q_{N}\left(g\right)+\lambda R\left(g\right),$$

with a penalty term expressed by a non-negative regularized R(g), and a regularization parameter λ>0 has to equilibrate both terms of (10) in an appropriate manner [22]. We make the following assumption:

**Assumption** **7.** *The regularizer R(g) is a continuous, differentiable strictly positive Lipchitz function.*

Note that for the regularizers,

(11) $$R\left(g\right)={\left(g-g_{0}\right)}^{T}W\left(g-g_{0}\right),$$

where $g_{0}\in G$ typically unifies all available a priori information on the optimal model parameter [23], and W is positive definite weight matrix, as well as

(12) $$R\left(g\right)=g^{T}Wg$$

which implies the parameter of the least (weighted) norm, Assumption 7, is satisfied.

Then, for fixed regularization parameter λ, the extremal problem

(13) $$\underset{g\in G}{\min}\;\;L_{N}\left(g,\lambda \right)=L_{N}\left(g_{N}^{\lambda },\lambda \right).$$

must be solved to obtain the optimal regularized model parameter $g_{N}^{\lambda }$. However, the minimum $g_{N}^{\lambda }$ can be not unique. Let the set of vectors $g_{N}^{\lambda }$ that solve the optimization task (13) be denoted by $G_{N}^{\lambda }$. The regularized integral index (5) takes the form

(14) L(g,λ)=Q(g)+λR(g).

Let the set of model parameters $g^{*\lambda }$ that solve the task

(15) $$\underset{g\in G}{\min}\;\;L\left(g,\lambda \right)=L\left(g^{*\lambda },\lambda \right),$$

be denoted by $G^{*\lambda }$.

The results of identification, both the model parameters $g_{N}^{\lambda }$ and the resulting relaxation modulus $G_{M}\left(t,g_{N}^{\lambda }\right)$, are dependent on the measurement data, in particular on the sampling instants $T_{i}$.

## 3. Results and Discussion

In this section, the analysis of the asymptotical (when the number of measurements tends to infinity) properties of the optimal regularized model is conducted. The rate of the convergence of the optimal identified model to the optimal model, which does not depend on the measurement data, is analyzed. The choice of the regularization parameter for the nonlinear Tikhonov regularization scheme is discussed, and the guaranteed model approximation rule is discussed. The resulting identification algorithm is described, and the applicability of the concept of the sampling-instant-independent identification to two classes of polymer relaxation modulus models is analyzed. Next, the compactness assumption concerning the set of admissible model parameters, which are natural in the context of viscoelastic models of polymers and convenient when analyzing the mathematical properties of the method, will be weakened by omitting the upper parameter constraints, which simplifies the numerical search for the optimal model parameters. Finally, the analytical properties of the presented identification method are verified by numerical studies. It has been assumed that the “real” material is described by a bimodal Gauss-like relaxation spectrum, which is often used to describe the rheological properties of various polymers [15,16,17] and polymers used in food technology [18,21,22]. Generalized Maxwell and KWW models are determined using the noise-corrupted data from the randomized experiment. Both the asymptotic properties and the influence of the measurement noises on the solution have been studied.

### 3.1. Convergence Analysis

Now, we wish to investigate the stochastic-type asymptotic properties of the regularized approximation task given by Equation (13). Since for any λ>0

$$\left[L\left(g,\lambda \right)+\sigma^{2}\right]-L_{N}\left(g,\lambda \right)=Q\left(g\right)+\sigma^{2}-Q_{N}\left(g\right),$$

and, by (7), the expected value

(16) $$E\left[L_{N}\left(g,\lambda \right)\right]=L\left(g,\lambda \right)+\sigma^{2},$$

Property 2 from [14] directly implies the next proposition.

**Proposition** **1.** *Let λ>0. When Assumptions 1–7 are satisfied, then*

(17) $$\underset{g\in G}{\sup}\;\;\left|\left[L\left(g,\lambda \right)+\sigma^{2}\right]-L_{N}\left(g,\lambda \right)\right|\to 0\;\;w.p.1\;\;\mathrm{as}\;\;\;N\to \infty ,$$

where w.p.1 means “with probability one”.

The result (17) means that the regularized identification index $L_{N}\left(g,\lambda \right)$ (10) is arbitrarily close, uniformly in g over the set G, to its expected value, c.f., Equation (16).

Now we can proceed to the main results. Proposition 1 enables us to relate the relaxation modulus model parameter $g_{N}^{\lambda }$, solving the regularized task expressed by Equation (13) for the empirical index $Q_{N}\left(g\right)$ to the parameter $g^{*\lambda }$ that minimize the regularized deterministic function Q(g) in the optimization task (15). Namely, from the uniform in g∈G convergence of the regularized index $L_{N}\left(g\right)$ in Equation (17) for any λ>0, we conclude immediately the following, c.f., Assertion in [14] or Equation (3.5) in [13]:

**Proposition** **2.** *Let λ>0. Assume that Assumptions 1–7 are in force, $T_{1},\ldots ,T_{N}$ are independently and randomly selected from T, each according to probability distributions with density ρ(t). If the minima of the optimization tasks (15) and (13) are unique, then*

(18) $$g_{N}^{\lambda }\to g^{*\lambda }\;\;w.p.1\;\;\mathrm{as}\;\;N\to \infty$$

*and*

(19) $$G_{M}\left(t,g_{N}^{\lambda }\right)\to G_{M}\left(t,g^{*\lambda }\right)\;\;w.p.1\;\;\mathrm{as}\;\;N\to \infty .$$

*for all t∈T. If the parameters solving the optimization tasks (15) and (13) are not unique, then for any convergent subsequence $\left\{g_{N}^{\lambda }\right\}$ with $g_{N}^{\lambda }\in G_{N}^{\lambda }$,*

(20) $$g_{N}^{\lambda }\to G^{*\lambda }\;w.p.1\;\;\mathrm{as}\;\;N\to \infty$$

*and for all t∈T and some $g^{*\lambda }\in G^{*\lambda }$, the asymptotic property (19) holds.*

By the compactness of G (Assumption 2), for any λ>0, the existence of a convergent subsequence $\left\{g_{N}^{\lambda }\right\}$ so that (20) holds is guaranteed.

Thus, for any fixed λ>0, under the taken assumptions, the regularized parameter $g_{N}^{\lambda }$ of the relaxation modulus model is a strongly consistent estimate of some parameter $g^{*\lambda }\in G^{*\lambda }$. Moreover, since the model $G_{M}\left(t,g\right)$, g∈G is Lipschitz on G uniformly in t∈T, then the almost-sure convergence of $g_{N}^{\lambda }$ to the respective parameter $g^{*\lambda }$ in Equation (18) implies that, c.f., ([14]: Remark 2):

(21) $$\underset{t\in T}{\sup}\;\;\left|G_{M}\left(t,g_{N}^{\lambda }\right)-G_{M}\left(t,g^{*\lambda }\right)\right|\to 0\;\;w.p.1\;\;\mathrm{as}\;\;N\to \infty .$$

i.e., that $G_{M}\left(t,g_{N}^{\lambda }\right)$ is a strongly uniformly consistent estimate of the best model $G_{M}\left(t,g^{*\lambda }\right)$ in the assumed class of models defined by Equation (2) for g∈G.

Summarizing, when Assumptions 1–7 are satisfied, the arbitrarily precise approximation of the optimal relaxation modulus model (with the regularized parameter $g^{*\lambda }$) can be obtained (almost everywhere) as the number of mesurements N grows large, despite the fact that the real description of the relaxation modulus is completely unknown.

### 3.2. Rate of Convergence

Taking into account the convergence in Equations (18) and (20), the question immediately arises of how fast $g_{N}^{\lambda }$ tends to some $g^{*\lambda }\in G^{*\lambda }$ as N grows large. The distance between the model parameters $g_{N}^{\lambda }$ and $g^{*\lambda }$ will be estimated in terms of the regularized integral identification index L(g,λ) (14), i.e., in the sense of the difference $\left|L\left(g^{*\lambda },\lambda \right)-L\left(g_{N}^{\lambda },\lambda \right)\right|$. We will examine how fast, for a given ε>0, the probability $P\left\{\left|L\left(g^{*\lambda },\lambda \right)-L\left(g_{N}^{\lambda },\lambda \right)\right|\geq \varepsilon \right\}$ tends to zero as N increases.

In Appendix A.1, for any ε>0 and any λ>0, using the well-known Hoeffding’s inequality [24], the following upper bound is derived:

(22) $$P\left\{\left|L\left(g_{N}^{\lambda },\lambda \right)-L\left(g^{*\lambda },\lambda \right)\right|\geq \varepsilon \right\}\leq 2\exp\left(\frac{-N\varepsilon^{2}}{8{\hat{M}}^{2}}\right),$$

where

(23) $$\hat{M}=\tilde{M}+\sigma^{2}+\delta^{2}+2\left(M+M_{3}\right)\delta ,$$

with some positive constant $\tilde{M}$ defined through inequality (A4) and the constants M and $M_{3}$ defined in Assumption 1 and Equation (3), respectively.

The inequality (22) shows connections between the convergence rate and the number of measurements N and the measurement noises. In particular, if ε is fixed, then the bounds for $P\left\{\left|L\left(g_{N}^{\lambda },\lambda \right)-L\left(g^{*\lambda },\lambda \right)\right|\geq \varepsilon \right\}$ tend to zero at an exponential rate as N increases. The rate of convergence is higher the lower are $\tilde{M}$, δ and $\sigma^{2}$, defined through inequality (A4) and Assumptions 5 and 6, respectively, i.e., the measurement noises are weaker. Additionally, analyzing (22), it is easy to see that stronger measurement disturbances reduce the convergence rate. The decrease in the speed is greater the larger that δ and σ are. This is not a surprise, since with large noises, the measurements are not very adequate compared to the true relaxation modulus. Notice, however, that for a fixed ε>0,

$$P\left\{\left|L\left(g_{N}^{\lambda },\lambda \right)-L\left(g^{*\lambda },\lambda \right)\right|\geq \frac{\varepsilon }{N^{\gamma }}\right\}\leq 2\exp\left(\frac{-N^{\left(1-2\gamma \right)}\varepsilon^{2}}{8{\left[\tilde{M}+\sigma^{2}+\delta^{2}+2\left(M+M_{3}\right)\delta \right]}^{2}}\right),$$

with $0\leq \gamma <\frac{1}{2}$ still tending to zero as N tends to infinity at a quasi-exponential rate.

### 3.3. Choice of the Regularization Parameter

Tikhonov regularization has been investigated extensively, both for the solution of linear as well as nonlinear ill posed problems. (see [11,12,22] for a survey on continuous regularization methods and references therein). For the minimization of the Tikhonov functional $Q_{N}\left(g\right)+\lambda R\left(g\right)$ for nonlinear ill-posed problems, usually, iterative methods are used. In [22], the regularization schemes based on different iteration methods, e.g., nonlinear Landweber iteration, level set methods, multilevel methods and Newton type methods, are presented. An analysis of the convexity of the Tikhonov functional, which guarantees global convergence of a wild class of numerical methods, has been carried out by Chavent and Kunisch [25,26].

There are different ways to decide on a suitable choice of the regularization parameter λ [12,27]. Here, we apply the guaranteed model approximation rule, which does not depend on a priori knowledge about the noise variance. The idea of this rule was first applied by Stankiewicz [28] for the identification of the relaxation time spectrum, and next, it has been successfully used for the Maxwell model identification task [29].

#### Guaranteed Model Approximation (GMA) Rule

Suppose that ${\bar{g}}_{N}$ is the optimal model parameter (usually not unique) minimizing the original empirical model approximation index $Q_{N}\left(g\right)$ (8) without regularization. The GMA technique when applied to a regularized task (13) relies on choosing as the regularization parameter the $\hat{\lambda }$ for which the assumed quality ${\hat{Q}}_{N}$ of the model approximation index so that ${\hat{Q}}_{N}>Q_{N}\left({\bar{g}}_{N}\right)$ is achieved for the minimizer $g_{N}^{\lambda }$ in the regularized optimization task (13), i.e.,

(24) $$Q_{N}\left(g_{N}^{\hat{\lambda }}\right)={\hat{Q}}_{N}.$$

Thus, as a result, the vector of the optimal regularized model parameters $g_{N}^{\hat{\lambda }}$ is determined. This rule is a quite natural strategy in the context of the relaxation modulus model approximation task, since the value of the mean-squares index (8) is directly taken into account. A certain interpretation of the GMA rule is given by the following result: for the proof, see Appendix A.2.

**Proposition** **3.** *Assume ${\hat{Q}}_{N}>Q_{N}\left({\bar{g}}_{N}\right)$. The regularized solution $g_{N}^{\hat{\lambda }}$, defined by (24), is the solution of the following optimization task*

(25) $$\underset{g\in G}{\min}\;\;R\left(g\right)\;\;\mathrm{under}\;\mathrm{the}\;\mathrm{constraint}\;Q_{N}\left(g\right)\leq {\hat{Q}}_{N}.$$

By Theorem 1, the GMA rule, Equation (24), relies on such a selection of the relaxation modulus model that the regularizer for the parameter $g_{N}^{\hat{\lambda }}$ is the smallest among all admissible models, so that $Q_{N}\left(g\right)\leq {\hat{Q}}_{N}$. Therefore, the best smoothness (in the sense of the assumed regularizer R(g)) of the model parameter vector $g_{N}^{\hat{\lambda }}$ is achieved. The effectiveness of this approach in the context of Maxwell model identification has been verified by the early paper [29], where the functional $L_{N}\left(g,\lambda \right)$ (10) with the quadratic regularizer $R\left(g\right)={\left\|g\right\|}_{2}^{2}$ was applied in the regularized optimization task (13).

### 3.4. Identification Algorithm

Taking into account the convergence results (18), (19) the calculation of the approximate value $g_{N}^{\lambda }$ of the optimal relaxation modulus model’s regularized parameter $g^{*\lambda }$ involves the following steps:

1. Choose the non-negative definite regularizer R(g) to regularize identification index $Q_{N}\left(g\right)$ (8).
2. Select randomly from the set T the sampling instants $t_{1},\ldots ,t_{N}$, selecting each $t_{i}$ independently, according to the probability distribution on T, with the density given by the weight function ρ(t) in the integral index Q(g) (5).
3. Perform the stress relaxation test [1,2,17] and record and store the relaxation modulus measurements $\left\{{\bar{G}}_{i}\right\}$, i=1,…,N, corresponding to the chosen points $t_{i}$.
4. Solve the regularized optimization task (13), applying the rule selected for choosing the regularization parameter, and compute the regularized model parameter $g_{N}^{\hat{\lambda }}$ for the chosen regularization parameter $\hat{\lambda }$.
5. Put $\bar{N}=N$ and $g_{\bar{N}}^{\hat{\lambda }}=g_{N}^{\hat{\lambda }}$. Select new $N\gg \bar{N}$ to enlarge the set of experimental data.
6. For new the N, repeat Steps 2, 3 and 4, i.e., select new sampling instants, perform again the stress relaxation test for the next sample of the same material and compute the new model parameter $g_{N}^{\hat{\lambda }}$.
7. In order to ascertain if $g_{\bar{N}}^{\hat{\lambda }}$ is a satisfactory approximation of $g^{*\hat{\lambda }}$, examine if ${\left\|g_{\bar{N}}^{\hat{\lambda }}-g_{N}^{\hat{\lambda }}\right\|}_{2}<\varepsilon$ for ε being a small positive number. If not, go again to Step 5. Otherwise, stop the procedure, taking $g_{\bar{N}}^{\hat{\lambda }}$ as the approximate value of $g^{*\hat{\lambda }}$.

**Remark** **1.** *The stopping rule from step 7 can be replaced by a less restrictive one, based on testing whether $\left|Q_{\bar{N}}\left(g_{\bar{N}}^{\hat{\lambda }}\right)-Q_{N}\left(g_{N}^{\hat{\lambda }}\right)\right|<\varepsilon$ holds. Both the considered stopping rules correspond with those that are commonly used in numerical minimization techniques.*

**Remark** **2.** *Note that no other special assumptions about $\left\{t_{i}\right\}$ were made.*

**Remark** **3.** *The relaxation modulus is the stress induced in the material when the unit’s step strain is imposed. However, loading is never performed infinitely fast [30,31]. Therefore, the relaxation modulus must be recovered from the experimental data of the stress relaxation process history, collected in non-ideal two-phase stress relaxation tests, where the strain increases over the loading time interval until a predetermined strain is reached, after which the strain is held constant. From different methods proposed for the relaxation modulus determination [30,32,33,34], the backward recursive method developed by Lee and Knauss [30], the differential rule proposed by Sorvari and Malinen [32], and the general method proposed by Zapas and Phillips [33] are most often cited. For detailed references and an overview, see [31,34].*

**Remark** **4.** *The regularization parameter $\hat{\lambda }$ is selected only once in step 4 and used for the next sets of measurements.*

**Figure 1:**
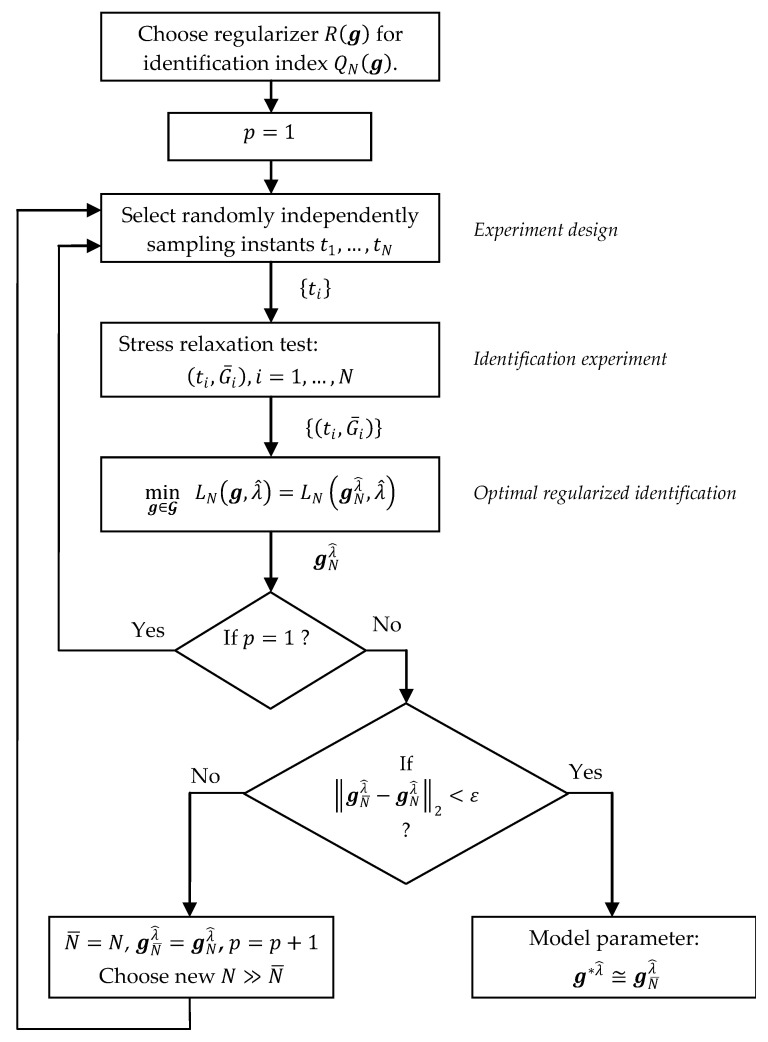
The flow chart of the algorithm for the relaxation modulus model identification.

The schematic framework of the above identification procedure and the communication between the regularized optimization tasks and the relaxation test experiment are shown in Figure 1. Based on Proposition 3, the optimization task (25) was applied in Step 4 to determine the optimal parameter $g_{N}^{\hat{\lambda }}$. The additional variable p is the index of the subsequent repetition of the stress relaxation experiment.

### 3.5. Applicability to Polymer Relaxation Modulus Models

The exponential Maxwell model and the exponential stretched Kohlrausch–Williams–Watts model are the best known linear rheological models of polymers [35]. To approximate nonexponential relaxation, inverse power laws were also used [36,37], especially the fractional Scott–Blair model [38]. Fractional viscoelasticity, described for example by the fractional Maxwell model, appears to be an appealing tool to describe the relaxation processes in polymers exhibiting both exponential and nonexponential types [38]. However, the applicability of the idea of identification that is asymptotically independent of the time instants used in the stress relaxation experiment to the fractional order model determination will be the subject of a separate paper.

The generalized Maxwell model, with relaxation modulus described by a linear combination of exponential terms, is still one of the most widely used rheological models of polymers. Application examples from just the last few years include studies on the long-term behavior of semi-crystalline bio-based fibers [39]; modeling the stress relaxation in stress-induced polymer crystallization [40]; a description of the stress relaxation after low- and high-rate deformation of polyurethanes [41]; and studying viscoelastic properties of hydroxyl-terminated polybutadiene (HTPB)-based composite propellants [42]. The Maxwell model has been successfully applied to stress relaxation predictions of many polymer composites [5]. For example, to the modeling of the viscoelastic properties of barium titanate (BTO)-elastomer (Ecoflex) composites [43]; modern photocurable MED610 resin, which is used mainly in medicine and dentistry [44]; perfluorosulfonic acid-based materials (applied in proton exchange membrane fuel cells) [45]; and the viscoelastic behavior of virgin EPDM/reclaimed rubber blends [46].

The Kohlrausch–Williams–Watts (KWW) relaxation function has been widely used to describe the relaxation behavior of glass-forming liquids and complex systems [47]. However, in the ongoing debate on the application of the KWW function to relaxation phenomena in different polymers [48,49], in particular to liquids and glasses, this model is widely used. Examples of research published this year alone include modeling of the mechanical properties of highly elastic and tough polymer binders with interweaving polyacrylic acid (PAA) with a poly(urea-urethane) (PUU) elastomer [50]; modeling of the relaxation curve describing the local dynamics of ions and hydration water near the RNA interface [51]; studying the relaxation processes for bulk antipsychotic API aripiprazole (APZ) and the active pharmaceutical ingredients (API) incorporating anodic aluminum oxide (AAO) or silica (SiO_2_) systems that are collected during the “slow-heating” procedure [52]; description of the rheological properties of the cross-linked blends of Xanthan gum and polyvinylpyrrolidone-based solid polymer electrolyte [53]; studies concerning the stress relaxation process in annealed metallic and polymer glasses [54]; and the stress relaxation behavior of glass-fiber-reinforced thermoplastic composites [55].

Although these models are continuous and differentiable with respect to the parameters (Assumption 3) and although the parameters are positive or non-negative due to the physical interpretation, the satisfaction of Assumptions 2–5, which are related to the models and the sets of their admissible parameters, is not obvious. We analyze them separately for the two classes of models.

#### 3.5.1. Generalized Maxwell Model

**Figure 2:**
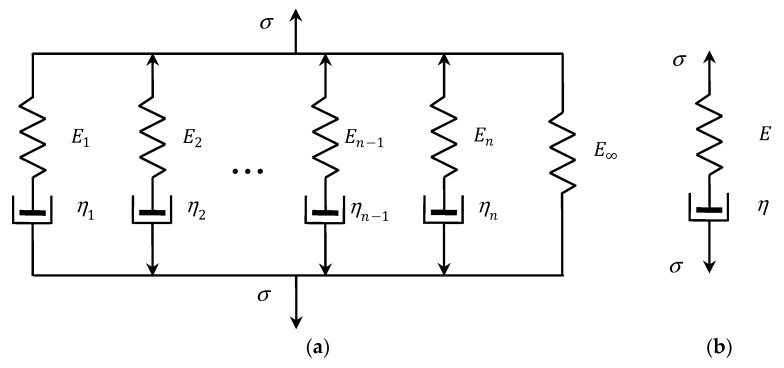
(**a**) Generalized Maxwell model; (**b**) a Maxwell unit; elastic modulus E, $E_{i}$, $E_{\infty }$ and viscosities η, $\eta_{i}$, i=1,…,n.

The generalized discrete Maxwell model, which is used to describe the relaxation modulus G(t) of linear viscoelastic materials, consists of a spring and n Maxwell units that are connected in parallel, as illustrated in Figure 2a. A Maxwell unit is a series arrangement of Hooke and Newton’s elements: an ideal spring in a series with a dashpot, c.f., Figure 2b. This model presents a relaxation of an exponential type given by a finite Dirichlet–Prony series [3]:

(26) $$G_{M}\left(t,g\right)=\sum_{j=1}^{n}E_{j}e^{-tv_{j}}+E_{\infty },$$

with the vector of the model parameters defined as

(27) $$g={\left[\begin{array}{ccccccc} E_{1} & \cdots & E_{n} & v_{1} & \cdots & v_{n} & E_{\infty } \end{array}\right]}^{T},$$

where $E_{j}\geq 0$, $v_{j}\geq 0$ and $E_{\infty }\geq 0$ are the parameters representing the elastic modulus (relaxation strengths), relaxation frequencies and equilibrium modulus (long-term modulus), respectively. The elastic modulus $E_{j}$ and the partial viscosity $\eta_{j}$ associated with the j Maxwell mode determine the relaxation frequency $v_{j}=E_{j}/\eta_{j}$ and the relaxation time $\tau_{j}=\eta_{j}/E_{j}$. The restriction that these parameters are non-negative and bounded must be given to satisfy the physical meaning. Thus, $g\in G\subset {ℛ}_{+}^{2n+1}$, where G is an arbitrary compact subset of ${ℛ}_{+}^{2n+1}$.

For the function $G_{M}\left(t,g\right)$ (26), for any t∈T, we have $G_{M}\left(t,0_{2n+1}\right)=0$, and by Equations (26) and (27), the gradient

$$\nabla_{g}G_{M}\left(t,g\right)={\left[\begin{array}{ccccccc} e^{-tv_{1}} & \cdots & e^{-tv_{n}} & -E_{1}te^{-tv_{1}} & \cdots & -E_{n}te^{-tv_{n}} & 1 \end{array}\right]}^{T}.$$

In view of the boundness of the function $te^{-tv_{i}}$ for any t∈T (whether T is bounded or not), Assumptions 2–4 are satisfied for any g∈G, provided that G is a compact subset of the subspace ${ℛ}_{+}^{2n+1}$.

By (26), the following inequalities hold

$$G_{M}\left(t,g\right)\leq \sum_{j=1}^{n}\left|E_{j}\right|+\left|E_{\infty }\right|\leq \sum_{j=1}^{n}\left|E_{j}\right|+\sum_{j=1}^{n}\left|v_{j}\right|+\left|E_{\infty }\right|,$$

whence, by virtue of the inequality ${\left\|x\right\|}_{1}\leq \sqrt{K}\;{\left\|x\right\|}_{2}$ [56] of the vector norm equivalence, where ${\left\|x\right\|}_{1}$ is the 1-norm (also called the Taxicab norm or Manhattan norm) in the space $R^{K}$, we immediately obtain the following property:

**Property** **1.** *For the relaxation modulus (26) of the Maxwell model, the estimation $G_{M}\left(t,g\right)\leq \sqrt{2n+1}{\left\|g\right\|}_{2}$ holds for an arbitrary t≥0 and arbitrary vector g (27) of non-negative model parameters.*

Property 1 and similar properties for subsequent models allow us to omit the upper constraints imposed on the model parameters in the optimization task (13) when the smoothing functions R(g) (11) or (12) are used.

#### 3.5.2. KWW Model

The exponential stretched Kohlrausch–Williams–Watts (KWW) model [8,9] describes the relaxation modulus as follows:

(28) $$G_{M}\left(t,g\right)=G_{0}e^{-{\left(v_{r}t\right)}^{\beta }},$$

where an adjustable parameter $G_{0}>0$ is the initial relaxation modulus, a stretching parameter 0<β≤1 is the exponent-spread factor, which quantitatively characterizes the non-Debye (nonexponential) character of the relaxation function, and $v_{r}>0$ and $\tau_{r}=1/v_{r}>0$ are, respectively, the characteristic relaxation time and frequency. Thus, the vector of the KWW model’s non-negative parameters is defined as

(29) $$g={\left[\begin{array}{ccc} G_{0} & v_{r} & \beta \end{array}\right]}^{T}.$$

For any t∈T function, $G_{M}\left(t,g\right)$ (28) is continuous and differentiable with respect to g; the gradient is as follows:

$$\nabla_{g}G_{M}\left(t,g\right)={\left[\begin{array}{ccc} e^{-{\left(v_{r}t\right)}^{\beta }} & -G_{0}\beta v_{r}^{-1}{\left(v_{r}t\right)}^{\beta }e^{-{\left(v_{r}t\right)}^{\beta }} & -G_{0}{\left(v_{r}t\right)}^{\beta }ln\left(v_{r}t\right)e^{-{\left(v_{r}t\right)}^{\beta }} \end{array}\right]}^{T},$$

and $G_{M}\left(t,0_{3}\right)=0$. The boundness of the first gradient element is obvious. By the inequality $e^{-x}\leq \frac{1}{1+x}$, that holds for any x>−1, we have

(30) $$G_{0}\beta v_{r}^{-1}{\left(v_{r}t\right)}^{\beta }e^{-{\left(v_{r}t\right)}^{\beta }}\leq G_{0}\beta v_{r}^{-1}\frac{{\left(v_{r}t\right)}^{\beta }}{1+{\left(v_{r}t\right)}^{\beta }}<G_{0}\beta v_{r}^{-1}.$$

The right constraint in (30) is bounded whenever the relaxation frequency $v_{r}\geq \tilde{\epsilon }$, where $\tilde{\epsilon }$ is a small positive constant. However, for any positive t and 0<β<1, the derivative $\partial G_{M}\left(t,g\right)/\partial v_{r}$ becomes unbounded when $v_{r}\to 0^{+}$. The third derivative $\partial G_{M}\left(t,g\right)/\partial \beta$ for any positive t and 0<β<1 tends to zero, by negative values, when $v_{r}t\to 0^{+}$ and is bounded for any bounded parameter g. Summarizing, the compact set of admissible model parameters is such that

$$G=\left\{g={\left[\begin{array}{ccc} G_{0} & v_{r} & \beta \end{array}\right]}^{T}:0\leq G_{0},0<\tilde{\epsilon }\leq v_{r},0\leq \beta \leq 1,{\left\|g\right\|}_{2}\leq M_{2}<\infty \right\},$$

for an arbitrary $M_{2}>\tilde{\epsilon }$. By assumption, a stretching parameter 0<β≤1; however, from the model identification point of view, it is convenient to expand this set to 0≤β≤1. For β=0, the relaxation modulus model is trivial, and $G_{M}\left(t,g\right)=G_{0}$ for any t, i.e., its boundness, differentiability and continuity are preserved.

The next property follows directly from the inequality $G_{M}\left(t,g\right)\leq G_{0}$, yielded by (28).

**Property** **2.** *For the relaxation modulus (28) of the KWW model, the estimation $G_{M}\left(t,g\right)\leq {\left\|g\right\|}_{2}$ holds for an arbitrary t≥0 and arbitrary vector g (29) of non-negative model parameters.*

### 3.6. Unconstrained Optimization

The compactness of the set of admissible model parameters G was significant for the convergence results (18)–(21). Due to the compactness of G, the existence of the optimal solutions to regularized tasks (13) and (15) is obvious. However, if the quadratic regularizers expressed by Equations (11) or (12) are applied, the upper constraints on the model parameters can be neglected, provided that they are not motivated by the parameters’ physical meaning. The following two lemmas are instrumental, proved in Appendix A.3 and Appendix A.4:

**Lemma** **1.** *Let λ>0. If Assumptions 1–3 and 6 hold, the weight matrix W of the regularizer R(g) (11) is a positive definite and the relaxation modulus model $G_{M}\left(t,g\right)$ (2) is such that $\left|G_{M}\left(t,g\right)\right|\leq p{\left\|g\right\|}_{2}$ for any t∈T, where 0<p<∞, then a compact subset of ${ℛ}^{K}$ exists:*

(31) $$\bar{G}=\left\{g\in {ℛ}_{+}^{K}:\;{\left\|g\right\|}_{2}\leq \bar{M}<\infty \right\},$$

*where*

(32) $$\bar{M}=\frac{2p\left(M+\delta \right)}{\lambda \;\lambda_{min}\left(W\right)}+\frac{2{\left\|Wg_{0}\right\|}_{2}}{\lambda_{min}\left(W\right)}>0,$$

*so that*

(33) $$\underset{g\in {ℛ}^{K}}{\min}\;\;Q_{N}\left(g\right)+\lambda R\left(g\right)=\underset{g\in \bar{G}}{\min}\;\;Q_{N}\left(g\right)+\lambda R\left(g\right),$$

*where $\lambda_{min}\left(W\right)>0$ is the minimal eigenvalue of W.*

**Lemma** **2.** *Let λ>0. If Assumptions 1–3 are satisfied, the weight matrix W of the regularizer R(g) (11) is a positive definite, and the relaxation modulus model $G_{M}\left(t,g\right)$ (2) is such that $\left|G_{M}\left(t,g\right)\right|\leq p{\left\|g\right\|}_{2}$ for any t∈T, where 0<p<∞, then a compact subset of ${ℛ}^{K}$ exists:*

(34) $$\bar{\bar{G}}=\left\{g\in {ℛ}_{+}^{K}:\;{\left\|g\right\|}_{2}\leq \bar{\bar{M}}<\infty \right\},$$

*where*

(35) $$\bar{\bar{M}}=\frac{2pM}{\lambda \;\lambda_{min}\left(W\right)}+\frac{2{\left\|Wg_{0}\right\|}_{2}}{\lambda_{min}\left(W\right)}>0,$$

*so that*

(36) $$\underset{g\in {ℛ}^{K}}{\min}\;\;Q\left(g\right)+\lambda R\left(g\right)=\underset{g\in \bar{\bar{G}}}{\min}\;\;Q\left(g\right)+\lambda R\left(g\right).$$

Letting $g_{0}=0_{K}$, we obtain a regularizer R(g) (12) instead of that expressed by (11), and therefore, the above lemmas hold also for the regularizer $R\left(g\right)=g^{T}Wg$ when applied during numerical studies.

Since the quality indices Q(g) and $Q_{N}\left(g\right)$ are continuous with respect to g, and the sets $\bar{\bar{G}}$ (34) and $\bar{G}$ (31) are compact in the space ${ℛ}^{K}$, in view of the above lemmas, for any λ>0 there exist the solutions to the upper-constrained regularized optimal approximation tasks given by the right hand sides of Equations (33) and (36) as well as the upper-unconstrained optimal approximation task expressed by the left hand sides of these equations. Since $\bar{\bar{M}}<\bar{M}$, further, we have $\bar{\bar{G}}\subset \bar{G}$. Therefore, both the optimization tasks (33) and (36) can be reduced to the set $\bar{G}$. If the previously defined set of admissible model parameters G is such that $\bar{G}\subset G$, then the upper constraints in the optimization tasks (15) and (13) can be neglected in view of Lemmas 1 and 2. If $\bar{G}$ is not a subset of G, then, for example, expanding the set of model parameters to $\bar{G}\cup G$ can be used to simplify numerical optimization tasks.

### 3.7. Numerical Studies

We now present the results of the numerical studies of the asymptotic properties of the identification algorithm and the influence of the measurement noises on the optimal model. In the context of an ill-posed problem, simulation studies allow us, apart from the theoretical analysis carried out above, to demonstrate the validity and effectiveness of the proposed identification method.

It is assumed that material viscoelastic properties are described by the double-mode Gauss-like relaxation spectrum. The Gaussian-like distributions were used to describe the rheological properties of many polymers, e.g., poly(methyl methacrylate) [16], polyethylene [15], native starch gels [20], polyacrylamide gels [17], glass [57] and carboxymethylcellulose [19]. The spectra of a Gaussian character were determined for bimodal polyethylene by Kwakye-Nimo et al. [15] and for soft polyacrylamide gels by Pérez-Calixto et al. [17]. Also, the spectra of various biopolymers determined by many researchers are Gaussian in nature, for example, cold gel-like emulsions stabilized with bovine gelatin [18], fresh egg white-hydrocolloids foams [19], some (wheat, potato, corn and banana) native starch gels [20], xanthan gum water solution [19], carboxymethylcellulose (CMC) [19], wood [58,59], and fresh egg white-hydrocolloids [19]. Three-, five- and seven-parameter Maxwell models (26) were determined. Next, the KWW model (28) was considered.

The “real” material and all the models were simulated in Matlab R2023b, using the special function *erfc* for the Gauss-like distribution.

### 3.8. Simulated Material

Consider viscoelastic material of a relaxation spectrum, described by the double-mode Gauss-like distribution considered in [15,60,61]:

$$ℋ\left(\tau \right)=\left[\vartheta_{1}e^{-{\left(\frac{1}{\tau }-m_{1}\right)}^{2}/q_{1}}+\vartheta_{2}e^{-{\left(\frac{1}{\tau }-m_{2}\right)}^{2}/q_{2}}\right]/\tau ,$$

inspired by polyethylene data from [15], especially the HDPE 1 sample from [15] (Table 1 and Figure 8b), where the parameters are as follows: [60,61]: $\vartheta_{1}=467\;\mathrm{Pa}\cdot s$, $m_{1}=0.0037{\;s}^{-1}$, $q_{1}=1.124261\times 10^{-6}{\;s}^{-2}$, $\vartheta_{2}=39\;\mathrm{Pa}\cdot s$, $m_{2}=0.045{\;s}^{-1}$ and $q_{2}=1.173\times 10^{-3}{\;s}^{-2}$. It is shown in [60] that the related real relaxation modulus is

(37) $$G\left(t\right)=\frac{\sqrt{\pi }}{2}\left[\vartheta_{1}\sqrt{q_{1}}\;e^{\frac{1}{4}t^{2}q_{1}-m_{1}t}erfc\left(\frac{\frac{1}{2}tq_{1}-m_{1}}{\sqrt{q_{1}}}\right)+\vartheta_{2}\sqrt{q_{2}}\;e^{\frac{1}{4}t^{2}q_{2}-m_{2}t}erfc\left(\frac{\frac{1}{2}tq_{2}-m_{2}}{\sqrt{q_{2}}}\right)\right].$$

Following [60,61], the time interval T=[0,1550] seconds is assumed for numerical simulations. Therefore, the weighting function in the index Q(g) (5) is $\rho \left(t\right)=\frac{1}{1550}{\;s}^{-1}$.

In the simulated stress relaxation experiment, N sampling instants $t_{i}$ were selected randomly according to the uniform distribution on T. Additive measurement noises $\left\{z_{i}\right\}$ were generated independently by random choice with a normal distribution, with zero mean value and variance $\sigma^{2}$. For the analysis of the asymptotic properties of the scheme N=50, 100, 250, 500, 1000, 2500, 5000, 10,000 has been used. In order to study the influence of the noises on the parameters of the optimal regularized models, the noises $\left\{z_{i}\right\}$ have been generated with the standard deviation σ=0.001, 0.004, 0.007 [Pa].

For every class of models (Maxwell, KWW) and for any pair (N,σ), the simulated experiment was performed. Next, for any class of models and any σ, the regularization parameter $\hat{\lambda }$ was selected according to the GMA rule, using the experiment data for the smallest N=50. For this purpose, the parameter vectors ${\bar{g}}_{N}$, minimizing the mean quadratic identification nonregularized index $Q_{N}\left(g\right)$ (8) in the optimization task (9), were determined for N=50. Through the inspection of the relation between elements of the vector ${\bar{g}}_{N}$, the diagonal positive definite weight matrices

(38) $$W=\mathrm{diag}\left(w_{11},w_{22},\ldots ,\;w_{KK}\right),$$

were selected for the regularizer R(g) (12). Next, the model approximation indices ${\hat{Q}}_{N}>Q_{N}\left({\bar{g}}_{N}\right)$ for the GMA rule (24) were assumed, and optimal regularization parameters $\hat{\lambda }$ related to the noises of the standard deviations σ=0.001, 0.004, 0.007 [Pa] were found, so that the GMA condition (24) holds. For successive models (three-, five- and seven-parameter Maxwell models and the KWW model), the vectors ${\bar{g}}_{N}$, indices $Q_{N}\left({\bar{g}}_{N}\right)$ and assumed ${\hat{Q}}_{N}$ and the regularization parameters $\hat{\lambda }$ are given in Table A1, Table A2, Table A3 and Table A4 in Appendix B. In the same tables, the elements of the vectors $g^{*\hat{\lambda }}$ which solve the optimization task (15) were presented, together with the related integral model approximation indices $Q\left(g^{*\hat{\lambda }}\right)$. In the last rows of these tables the diagonal elements $w_{kk}$, k=1, 2, 3, of the weight matrices W (38) were also given.

### 3.9. Asymptotic Properties

Then, for every class of models and any pair (N,σ), the optimal model parameter $g_{N}^{\hat{\lambda }}$ was determined by solving the regularized identification task (13) for $\lambda =\hat{\lambda }$. The elements of the parameter vectors $g_{N}^{\hat{\lambda }}$, the indices $Q_{N}\left(g_{N}^{\hat{\lambda }}\right)$ and $Q\left(g_{N}^{\hat{\lambda }}\right)$, as well as the relative percentage errors of the approximation of the measurement-independent parameters $g^{*\lambda }$, defined as

(39) $$ERR={\left\|g_{N}^{\hat{\lambda }}-g^{*\lambda }\right\|}_{2}^{2}/{\left\|g^{*\lambda }\right\|}_{2}^{2}\cdot 100\%,$$

are given in Table 1, Table 2, Table 3 and Table 4 for successive classes of models, and the weakest noises σ=0.001 [Pa].

**Figure 3:**
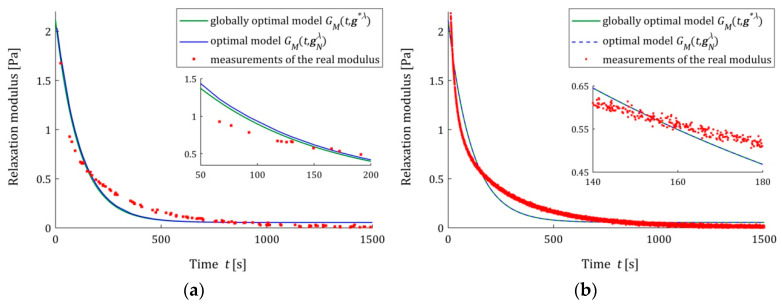
The real relaxation modulus measurements ${\bar{G}}_{i}$ (red points) and three-parameter Maxwell models: sampling-instant-independent model $G_{M}\left(t,g^{*\hat{\lambda }}\right)$ (green lines) and optimal models $G_{M}\left(t,g_{N}^{\hat{\lambda }}\right)$ (blue lines) for N measurements and additive random noises of normal distribution with zero mean value and standard deviation σ=0.007 Pa: (**a**) N=100; (**b**) N=10,000. The caret for $\hat{\lambda }$ has been omitted in the legend description to simplify it.

**Figure 4:**
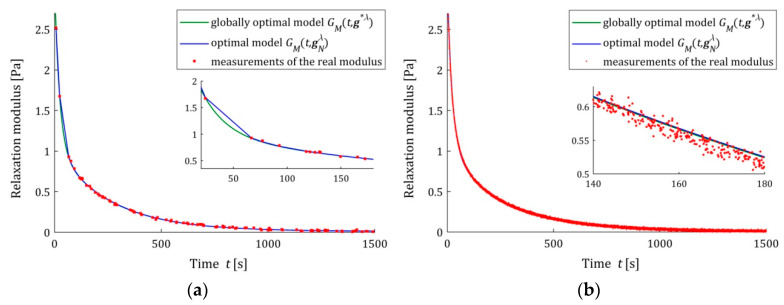
The real relaxation modulus measurements ${\bar{G}}_{i}$ (red points) and five-parameter Maxwell models: sampling-instant-independent model $G_{M}\left(t,g^{*\hat{\lambda }}\right)$ (green lines) and optimal models $G_{M}\left(t,g_{N}^{\hat{\lambda }}\right)$ (blue lines) for N measurements and additive random noises of normal distribution with zero mean value and standard deviation σ=0.007 Pa: (**a**) N=100; (**b**) N=10,000. The caret for $\hat{\lambda }$ has been omitted to simplify the legend description.

**Figure 5:**
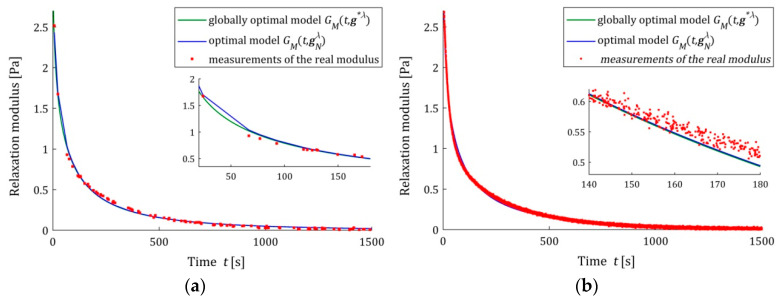
The real relaxation modulus measurements ${\bar{G}}_{i}$ (red points) and KWW models (28): sampling-instant-independent model $G_{M}\left(t,g^{*\hat{\lambda }}\right)$ (green lines) and optimal models $G_{M}\left(t,g_{N}^{\hat{\lambda }}\right)$ (blue lines) for N measurements and additive random noises of normal distribution with zero mean value and standard deviation σ=0.007 Pa: (**a**) N=100; (**b**) N=10,000. The caret for $\hat{\lambda }$ variable has been omitted to simplify the legend description.

The analysis of the identification indices $Q_{N}\left(g_{N}^{\hat{\lambda }}\right)$ and $Q\left(g_{N}^{\hat{\lambda }}\right)$ indicates that the five- and seven-parameter Maxwell models provide a much better approximation of the measurement data and the real relaxation modulus G(t) (37) than the three-parameter model and a better approximation than the KWW model. This is illustrated in Figure 3, Figure 4 and Figure 5, where for the successive classes of model measurements, the ${\bar{G}}_{i}$ of the real modulus G(t) fitted by the optimal models $G_{M}\left(t,g_{N}^{\hat{\lambda }}\right)$ are plotted for two numbers of measurements (N=100 and N=10,000) and the strongest noises. The plots for the seven-parameter Maxwell model being visually almost identical to that for the five-parameter model and providing an excellent data fit, especially for N=10,000 measurements, are omitted here; compare also indices $Q_{N}\left(g_{N}^{\hat{\lambda }}\right)$ and $Q\left(g_{N}^{\hat{\lambda }}\right)$ from Table 2 and Table 3. Although for N=100 measurements, the models $G_{M}\left(t,g_{N}^{\hat{\lambda }}\right)$ and $G_{M}\left(t,g^{*\hat{\lambda }}\right)$ differ in the initial time interval (see small subplots), for N=10,000 measurements, they are practically identical. This applies to the five-parameter Maxwell model with an almost excellent fitting (Figure 4b), as well as to the KWW (Figure 5b) and three-parameter Maxwell (Figure 3b) models with lower quality of the measurement data fit.

We see that also for the strongest noises, the model $G_{M}\left(t,g_{N}^{\hat{\lambda }}\right)$ tends to $G_{M}\left(t,g^{*\hat{\lambda }}\right)$ as N increases, even when the accuracy of the measurement data approximation is not excellent for a given class of models. Not only for N=10,000, but also for smaller numbers of measurements, these models coincide, which is confirmed by the ERR (39) values from Table 1, Table 2, Table 3 and Table 4. The relative percentage errors ERR (39) of the parameters $g^{*\lambda }$ and $g_{N}^{\hat{\lambda }}$ discrepancy is smaller than 0.1% for N≥5000 for the three-parameter Maxwell model with the worst fit to the measurement data and for N≥1000 for the KWW model. However, for more accurate, in terms of $Q_{N}\left(g_{N}^{\hat{\lambda }}\right)$ and $Q\left(g_{N}^{\hat{\lambda }}\right)$ indices, in the five- and seven-parameter Maxwell models, ERR (39) does not exceed for N≥100 measurements, respectively, 0.02% and 0.001%.

**Figure A1:**
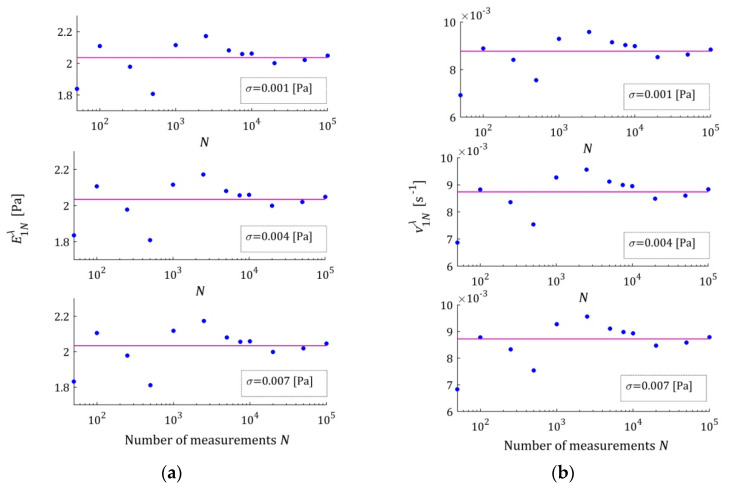

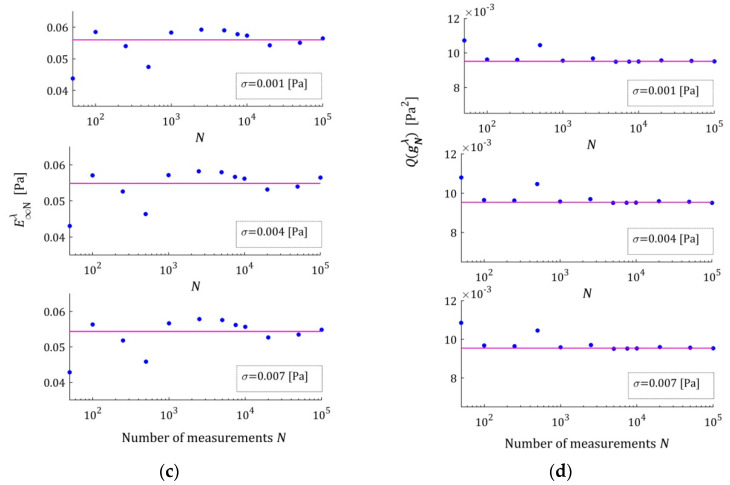
The optimal Maxwell model parameters: (**a**) $E_{1N}^{\hat{\lambda }}$; (**b**) $v_{1N}^{\hat{\lambda }}$; (**c**) $E_{\infty N}^{\hat{\lambda }}$; and (**d**) integral identification index $Q\left(g_{N}^{\hat{\lambda }}\right)$, Equation (5), as the functions of the number of measurements N for noises σ=0.001, 0.004, 0.007 [Pa]; the horizontal violet lines correspond to the elements $E_{1}^{*\hat{\lambda }}$, $v_{1}^{*\hat{\lambda }}$, $E_{\infty }^{*\hat{\lambda }}$ of the optimal regularized parameter $g^{*\hat{\lambda }}$ (subfigures (**a**–**c**)) and to the respective quadratic index $Q\left(g^{*\hat{\lambda }}\right)$ (subfigure (**d**)). The regularization parameters $\hat{\lambda }$ are given in Table A1. The caret under $\hat{\lambda }$ variable has been omitted to simplify the description of the plot axes.

**Figure A2:**
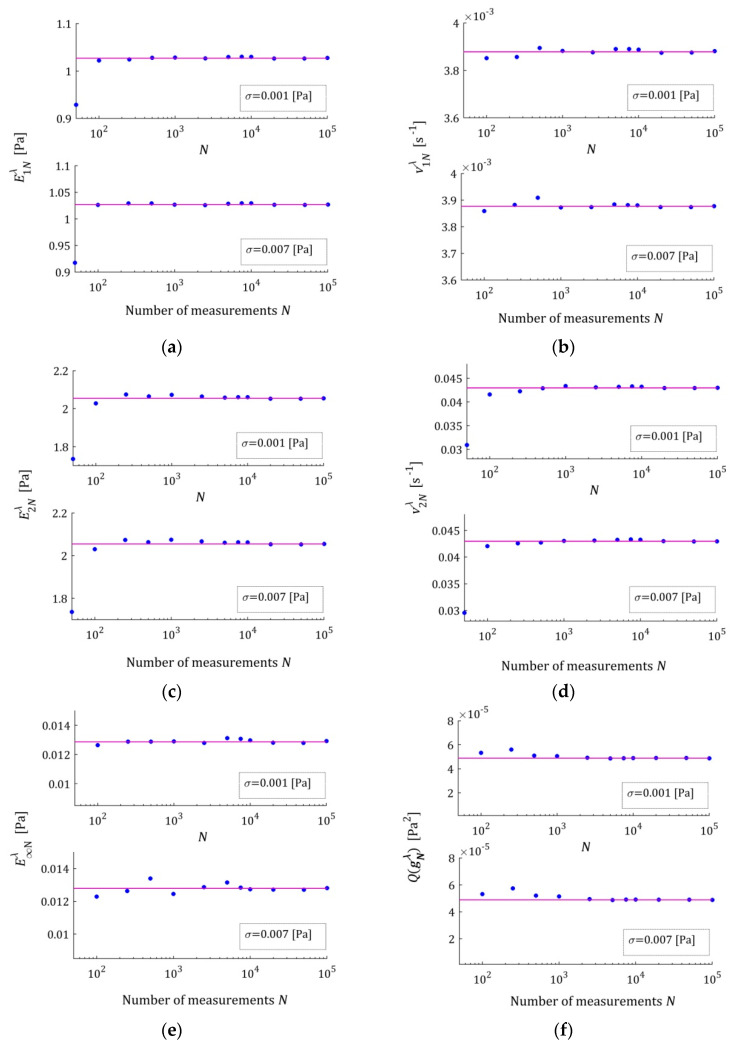
The optimal Maxwell model parameters: (**a**) $E_{1N}^{\hat{\lambda }}$; (**b**) $v_{1N}^{\hat{\lambda }}$; (**c**) $E_{2N}^{\hat{\lambda }}$; (**d**) $v_{2N}^{\hat{\lambda }}$; (**e**) $E_{\infty N}^{\hat{\lambda }}$; and (**f**) integral identification index $Q\left(g_{N}^{\hat{\lambda }}\right)$, Equation (5), as the functions of the number of measurements N for noises σ=0.001, 0.007 [Pa]; the horizontal violet lines correspond to the elements $E_{1}^{*\hat{\lambda }}$, $v_{1}^{*\hat{\lambda }}$, $E_{2}^{*\hat{\lambda }}$, $v_{2}^{*\hat{\lambda }}$ and $E_{\infty }^{*\hat{\lambda }}$ of the optimal regularized parameter $g^{*\hat{\lambda }}$ (subfigures (**a**–**e**)) and to the respective quadratic index $Q\left(g^{*\hat{\lambda }}\right)$ (subfigure (**f**)). The regularization parameters $\hat{\lambda }$ are given in Table A2. The caret for $\hat{\lambda }$ variable has been omitted to simplify the description of the plot axes.

**Figure A3:**
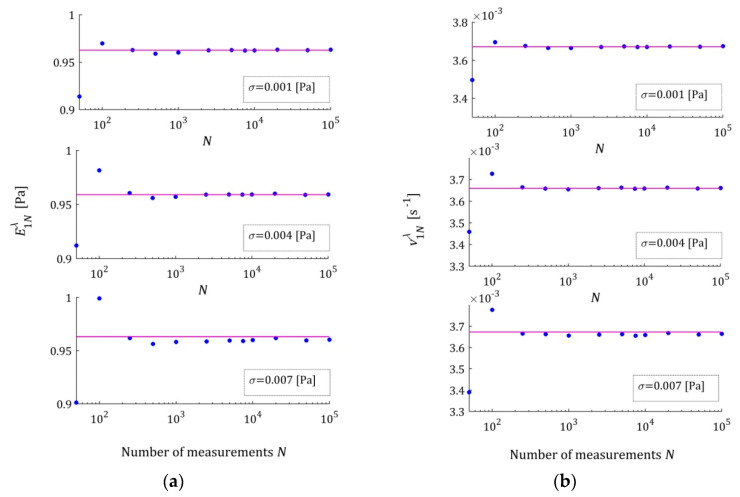

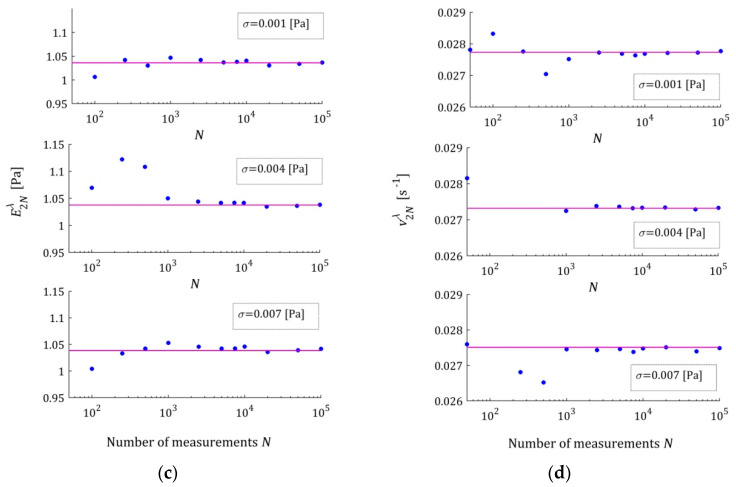
The parameters of the optimal seven-parameter Maxwell model: (**a**) $E_{1N}^{\hat{\lambda }}$; (**b**) $v_{1N}^{\hat{\lambda }}$; (**c**) $E_{2N}^{\hat{\lambda }}$; (**d**) $v_{2N}^{\hat{\lambda }}$ as the functions of the number of measurements N for noises σ=0.001, 0.004, 0.007 [Pa]; the horizontal violet lines correspond to the elements $E_{1}^{*\hat{\lambda }}$, $v_{1}^{*\hat{\lambda }}$, $E_{2}^{*\hat{\lambda }}$ and $v_{2}^{*\hat{\lambda }}$ of the optimal regularized parameter $g^{*\hat{\lambda }}$. The regularization parameters $\hat{\lambda }$ are given in Table A3. The caret for $\hat{\lambda }$ variable has been omitted to simplify the description of the plot axes.

**Figure A4:**
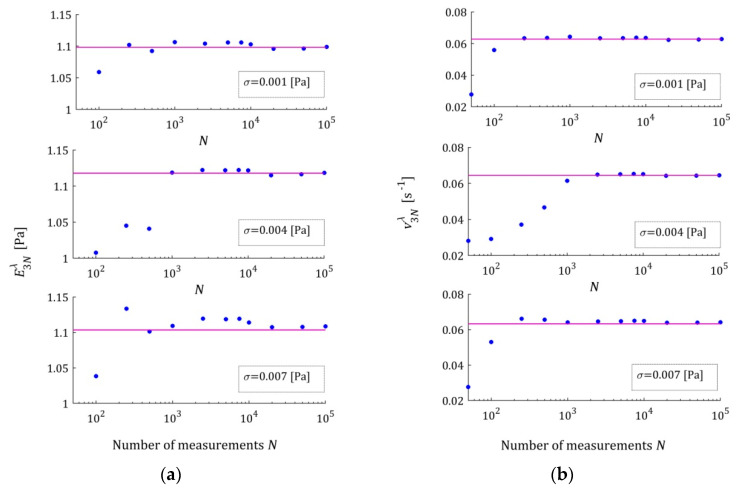

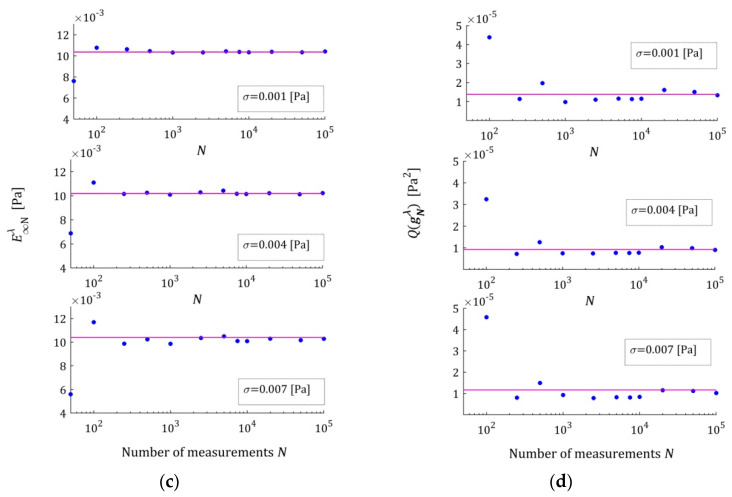
The parameters of the optimal seven-parameter Maxwell model: (**a**) $E_{3N}^{\hat{\lambda }}$; (**b**) $v_{3N}^{\hat{\lambda }}$; (**c**) $E_{\infty N}^{\hat{\lambda }}$; and (**d**) integral identification index $Q\left(g_{N}^{\hat{\lambda }}\right)$, Equation (5), as the functions of the number of measurements N for noises σ=0.001, 0.004, 0.007 [Pa]; the horizontal violet lines correspond to the elements $E_{3}^{*\hat{\lambda }}$, $v_{3}^{*\hat{\lambda }}$ and $E_{\infty }^{*\hat{\lambda }}$ of the optimal regularized parameter $g^{*\hat{\lambda }}$ (subfigures (**a**–**c**)) and the respective quadratic index $Q\left(g^{*\hat{\lambda }}\right)$ (subfigure (**d**)). The regularization parameters $\hat{\lambda }$ are given in Table A3. The caret for $\hat{\lambda }$ variable has been omitted to simplify the description of the plot axes.

**Figure A5:**
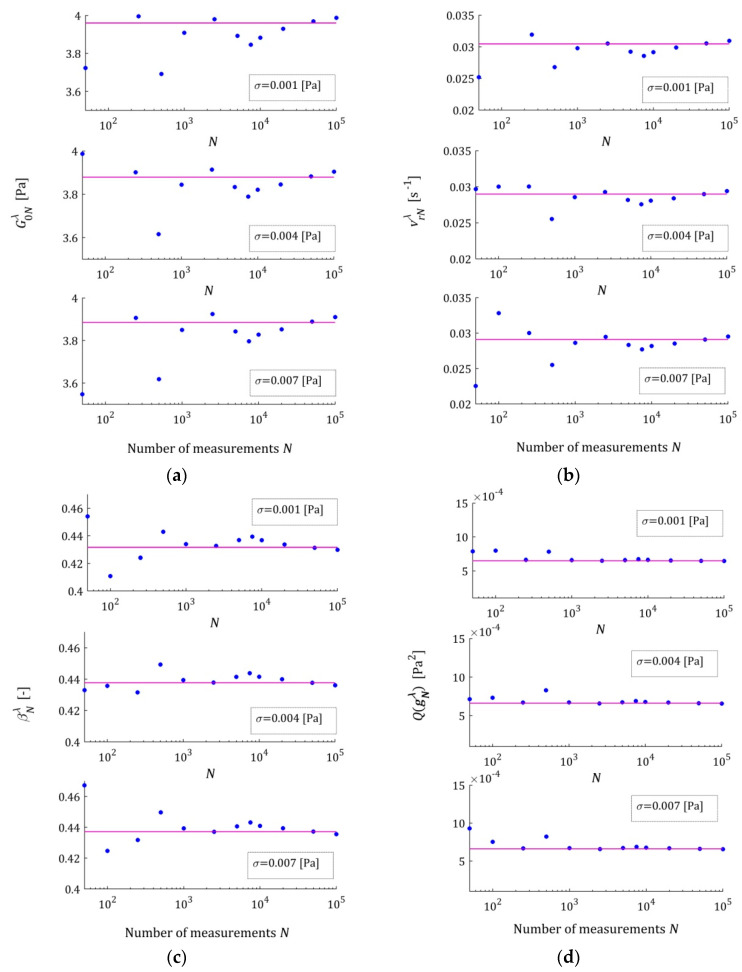
The optimal parameters of KWW model (28): (**a**) $G_{0N}^{\hat{\lambda }}$; (**b**) $v_{rN}^{\hat{\lambda }}$; (**c**) $\beta_{N}^{\hat{\lambda }}$; and (**d**) integral identification index $Q\left(g_{N}^{\hat{\lambda }}\right)$, Equation (5), as the functions of the number of measurements N for noises σ=0.001, 0.004, 0.007 [Pa]; the horizontal violet lines correspond to the elements $G_{0}^{*\hat{\lambda }}$, $v_{r}^{*\hat{\lambda }}$ and $\beta^{*\hat{\lambda }}$ of the parameter $g^{*\hat{\lambda }}$ (subfigures (**a**–**c**)) and to the respective quadratic index $Q\left(g^{*\hat{\lambda }}\right)$ (subfigure (**d**)). The regularization parameters $\hat{\lambda }$ are given in Table A4. The caret for $\hat{\lambda }$ variable has been omitted to simplify the description of the plot axes.

The dependence of the optimal model parameters $g_{N}^{\hat{\lambda }}$ on the number of measurements N for the noises of σ=0.001, 0.004, 0.007 [Pa] for the three-parameter Maxwell model are illustrated by Figure A1a–c in Appendix B.1. For the five-parameter Maxwell model, the elements of the optimal parameters $g_{N}^{\hat{\lambda }}$ as the functions of N are illustrated by Figure A2a–c, Appendix B.2. Only extreme—the strongest and weakest—disturbances are considered here to limit the size of the figure. For all the noises of σ=0.001, 0.004, 0.007 [Pa], the elements of $g_{N}^{\hat{\lambda }}$ for the seven-parameter Maxwell model are depicted in Figure A3 and Figure A4a–c in Appendix B.3, while for the KWW model, they are depicted in Figure A5a–c, Appendix B.4. In any subplot, the values of the respective parameters of the globally optimal model $g^{*\lambda }$ are plotted with horizontal violet lines. In these figures, a logarithmic scale is used on the horizontal axes. The asymptotic properties are also illustrated by Figure A1d, Figure A2d, Figure A4d and Figure A5d juxtaposing, for respective models, the integral index $Q\left(g_{N}^{\hat{\lambda }}\right)$ as a function of N with the index $Q\left(g^{*\hat{\lambda }}\right)$, marked with horizontal lines. In these figures, the caret for the $\hat{\lambda }$ variable has been omitted to simplify the description of the plot axes.

These plots confirm the asymptotic properties of the proposed identification algorithm. A better model fit, for the five- and seven-parameter Maxwell models $G_{M}\left(t,g_{N}^{\hat{\lambda }}\right)$, implies smaller fluctuations in the estimates of its parameters $g_{N}^{\hat{\lambda }}$ and their faster convergence to the parameters $g^{*\hat{\lambda }}$ of the sampling-point-independent model $G_{M}\left(t,g^{*\hat{\lambda }}\right)$. This property translates into the speed of the convergence $Q\left(g_{N}^{\hat{\lambda }}\right)$ into $Q\left(g^{*\hat{\lambda }}\right)$.

### 3.10. Noise Robustness

Now, we are interested in the noise robustness properties of the regularized identification algorithm when the concept of experiment randomization is applied to determine the standard relaxation modulus models.

To examine the impact of the measurement noises for every pair (N,σ), the experiment (simulated stress relaxation test) was repeated n=50 times, generating the measurement noises $\left\{z_{i}\right\}$ independently by random choice with a normal distribution, with zero mean value and variance $\sigma^{2}$.

Next, to estimate the approximation error of the relaxation modulus measurements for the n-element sample, the mean optimal relaxation modulus approximation error was determined:

(40) $$ERRQ_{N}=\frac{1}{n}\sum_{j=1}^{n}Q_{N}\left(g_{N,j}^{\hat{\lambda }}\right),$$

where $g_{N,j}^{\hat{\lambda }}$ is the vector of optimal model parameters determined for *j*-th experiment repetition for given pair (N,σ), j=1,…,n.

The mean optimal integral error of the true relaxation modulus approximation

(41) $$ERRQ=\frac{1}{n}\sum_{j=1}^{n}Q\left(g_{N,j}^{\hat{\lambda }}\right)$$

was also computed.

By generalization of the distance between the regularized vector of model parameters $g_{N,j}^{\hat{\lambda }}$ and the measurement-independent vector $g^{*\hat{\lambda }}$ (for noise-free measurements), estimated by relative error ERR (39), for the n element sample, the mean relative error of the parameter $g^{*\hat{\lambda }}$ approximation was defined as

(42) $$MERR=\frac{1}{n}\sum_{j=1}^{n}{\left\|g_{N,j}^{\hat{\lambda }}-g^{*\hat{\lambda }}\right\|}_{2}^{2}/{\left\|g^{*\hat{\lambda }}\right\|}_{2}^{2}\cdot 100\%.$$

**Figure 6:**
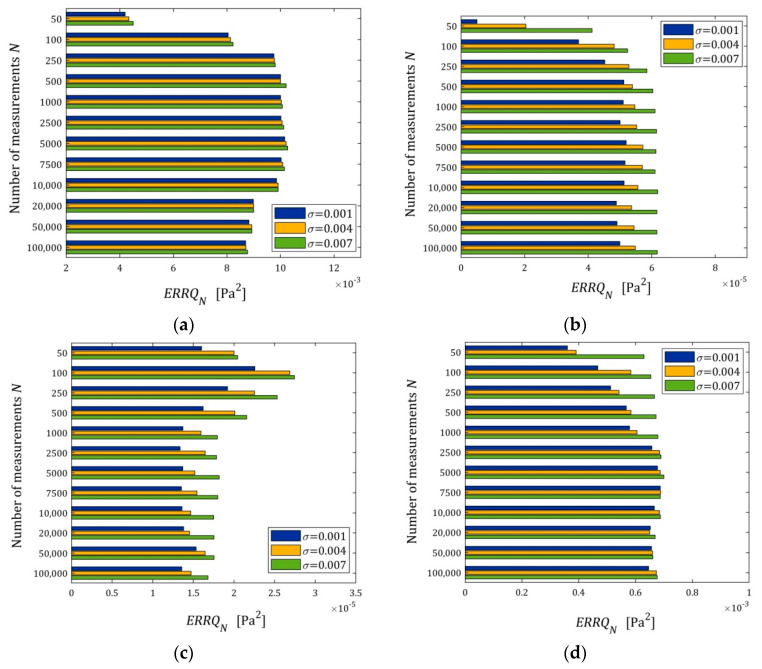
The mean empirical error $ERRQ_{N}$ (40) of the optimal relaxation modulus approximation as a function of the number of measurements N and the noise of standard deviation σ for: (**a**) three-; (**b**) five-; and (**c**) seven-parameter Maxwell models (26); (**d**) KKW Model (28).

The index $ERRQ_{N}$ (40) as a function of N and σ is depicted in the bar in Figure 6; linear scales are used for the index $ERRQ_{N}$ axis.

We can see that $ERRQ_{N}$ for N>100 does not depend essentially on the number of measurements, neither for small nor large noises. For the five- and seven-parameter Maxwell model, the algorithm ensures very good quality of the measurement approximation even for large noises, and for the three-parameter and KWW model, the measurement data fit is ten times weaker than for the five-parameter Maxwell model, but it is still a good approximation. For these models, with a poorer approximation quality, the impact of noises on the approximation quality is weaker than for the five- and seven-parameter Maxwell models, for which the approximation error comes primarily from measurement noises, especially for the seven-parameter model. However, for the five- and seven-parameter Maxwell models, the impact of noises on the approximation quality is slightly larger, and the indices of the order $10^{-5}÷10^{-4}$ are really very small.

**Figure 7:**
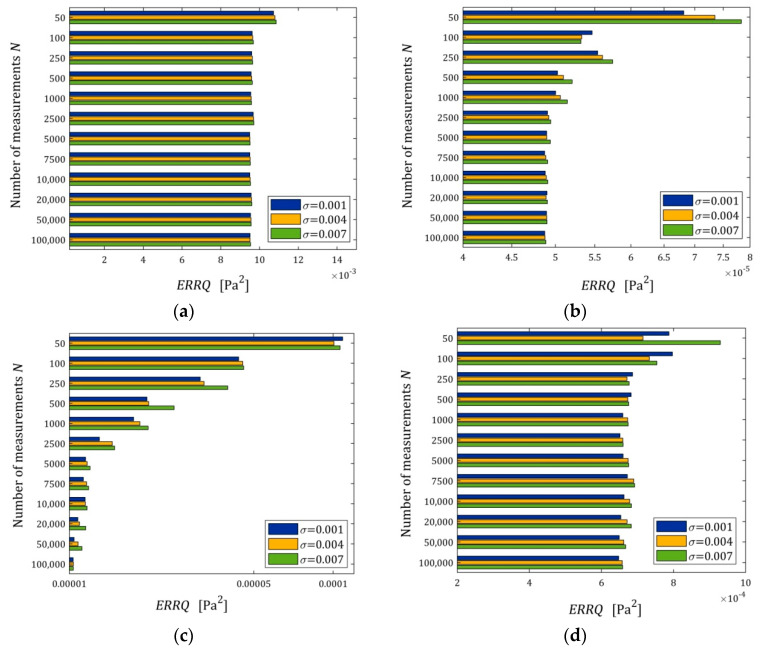
The mean optimal integral error of the true relaxation modulus approximation ERRQ (41) as a function of the number of measurements N and the noise of standard deviation σ for: (**a**) three-; (**b**) five-; and (**c**) seven-parameter Maxwell models (26); (**d**) KKW Model (28).

Figure 7 illustrates the dependence of the index ERRQ (41) on N and σ; for the three-parameter Maxwell model and the KWW model, linear scales are used for the ERRQ axis, while for the five- and seven-parameter Maxwell models, a logarithmic scale is applied.

The mean integral error ERRQ is, generally, a decreasing function of the number of sampling points and an increasing function of the noise standard deviation, as depicted in Figure 7. This is particularly visible in Figure 7c for the seven-parameter Maxwell model, which has an excellent fit to the measurement data, compare also Figure 6c. The interpretation of Figure 7c becomes quite clear when we take into account the convergence analysis conducted above. As we have shown, the global integral index Q(g) (strictly the function L(g,λ) (14)) converges exponentially both with the increase in the number of measurements N and with the decrease in the noise variance $\sigma^{2}$; compare the inequality in Equation (22) and the definition of $\hat{M}$ (23).

**Figure 8:**
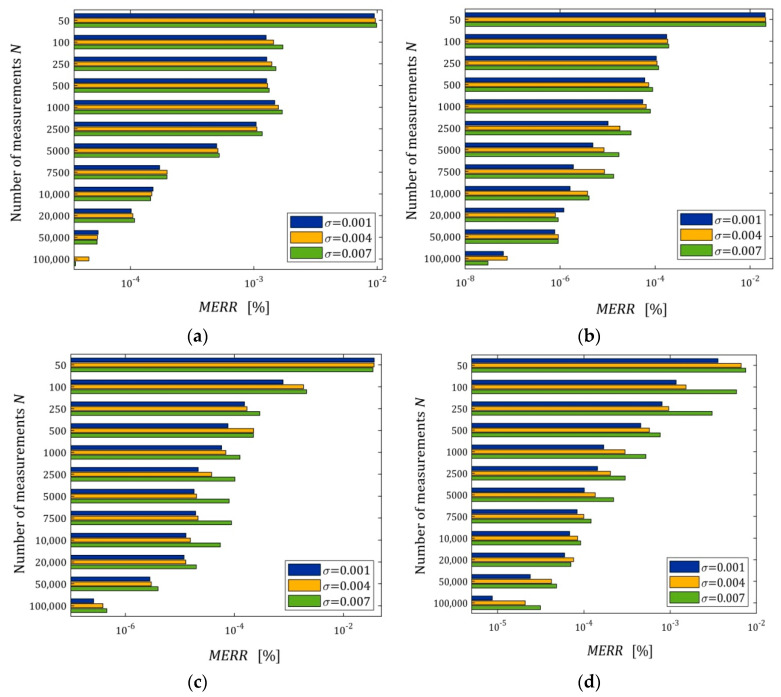
The mean elative error of the measurement-independent parameters $g^{*\lambda }$ approximation MERR (42) as a function of the number of measurements N and the noise of standard deviation σ for: (**a**) three-; (**b**) five-; and (**c**) seven-parameter Maxwell models (26); (**d**) KKW Model (28).

The relationships of the mean relative errors MERR (42) with N and σ are depicted in Figure 8; a logarithmic scale is used for the MERR axis in all subfigures. We can see that MERR decreases exponentially with the increasing number of measurements (logarithmic scale). This index is particularly small for more accurate models (five- and seven-parameter Maxwell models). It is of the order of $10^{-7}÷10^{-3}\;\%$ for N≥100 and even for the strongest disturbances, which practically means determining the globally optimal parameter $g^{*\lambda }$. The characteristics from Figure 8 also confirm the noise robustness of the estimators $g_{N}^{\hat{\lambda }}$ of the optimal parameter $g^{*\lambda }$, also for models that approximate the measurement data less well.

To sum up, not so much the dependence of the empirical index $ERRQ_{N}$ (40), but primarily the courses of the indices MERR (42) and ERRQ (41) as the functions of N, indicate the asymptotic independence of the model from the sampling points. The five- or even seven-parameter Maxwell models are necessary for an almost excellent fitting of the data, if the sampling time instants are chosen in an appropriate way.

## 4. Conclusions

The analytical analysis and numerical studies proved that an arbitrarily precise approximation of the optimal regularized relaxation modulus model that is independent of the sampling instants can be derived from relaxation modulus data sampled randomly according to respective randomization, when the number of the measurements applied in the stress relaxation test grows large, despite the fact that the real description of the relaxation modulus is completely unknown. The parameters of the approximate model are strongly consistent estimates of the parameters of the sampling-instants-independent model. The assumed conditions and restrictions are related primarily to a model, which can be selected to a certain extent, not to the real relaxation modulus.

The resulting identification procedure is very useful in application, because it does not require any other experimental technique that is more sophisticated than a priori independent random sampling of time instants from the assumed set according to a stationary rule. Therefore, the general statement that the choice of the sampling instants has fundamental meaning for the identified model finds the expected confirmation also in the context of polymer rheological models. Applying the scheme proposed, the three-, five- and seven-parameter Maxwell models and KWW models were determined, and the convergence of the sequence of the optimal parameters was demonstrated.

The relaxation modulus identification problem is not to achieve a true description of the real relaxation modulus, but one that is a “sufficiently or optimally accurate”. Modern computer-aided engineering design systems with an application of different polymers and different engineering plastics are combined with increasingly stronger applications of mathematical models and model-based numerical design methods. The more accurate and universal the model is, the better the design results may be. Therefore, an optimal sampling-instants-independent model provides more information for engineering design purposes than one that depends on the specific experiment data used in the experiment.

Fractional viscoelasticity, a new formalism introduced for the mathematical modeling of rheological materials, has been verified by many studies to be a solid tool to describe the relaxation processes in polymers exhibiting both exponential and nonexponential types. Fractional order models have gained research interest due to their improved flexibility compared with those offered by their classic, integer-order counterparts. However, the most known among them, the Blair–Scott and fractional Maxwell models, do not meet all the assumptions adopted here. The applicability of the idea of identification of the model, being asymptotically independent of the time instants used in the stress relaxation experiment, to the fractional order models determination will be the subject of future research.

The paper is concerned with the relaxation modulus modeling, but the proposed identification scheme can also be successfully applied to the identification of the creep compliance models, for example the Kelvin–Voigt model, using the measurements obtained in the creep test, whenever the respective set of sampling times is open to manipulation during the data collection.

## Figures and Tables

**Table 1:** The elements $E_{1N}^{\hat{\lambda }}$, $v_{1N}^{\hat{\lambda }}$ and $E_{\infty N}^{\hat{\lambda }}$ of the vector $g_{N}^{\hat{\lambda }}$ solving optimization task (13) for regularization parameter $\hat{\lambda }=0.0156$, the mean-square identification indices $Q_{N}\left(g_{N}^{\hat{\lambda }}\right)$, Equation (8), the integral quadratic indices $Q\left(g_{N}^{\hat{\lambda }}\right)$, Equation (5), and the relative square errors ERRs (39) of the measurement-independent parameters $g^{*\lambda }$, approximation for three-parameter Maxwell model (26) and N relaxation modulus measurements, corrupted by additive independent noises of normal distribution with standard deviation σ=0.001 [Pa].

N	$Q_{N}\left(g_{N}^{\hat{\lambda }}\right)\;\left[Pa^{2}\right]$	$Q\left(g_{N}^{\hat{\lambda }}\right)\;\left[Pa^{2}\right]$	ERR [%]	$E_{1N}^{\hat{\lambda }}\;\left[Pa\right]$	$v_{1N}^{\hat{\lambda }}\;\left[s^{-1}\right]$	$E_{\infty N}^{\hat{\lambda }}\;\left[Pa\right]$
50	4.2004 × 10^−3^	1.0723 × 10^−2^	0.9466	1.83859	6.92243 × 10^−3^	4.3772 × 10^−2^
100	8.0483 × 10^−3^	9.6203 × 10^−3^	0.1258	2.10863	8.89014 × 10^−3^	5.84914 × 10^−2^
250	9.7535 × 10^−3^	9.5998 × 10^−3^	8.123 × 10^−2^	1.97838	8.40937 × 10^−3^	5.39954 × 10^−2^
500	7.4646 × 10^−3^	1.0454 × 10^−2^	1.2699	1.80699	7.55342 × 10^−3^	4.74206 × 10^−2^
1000	1.0003 × 10^−2^	9.5483 × 10^−3^	0.1476	2.11463	9.29357 × 10^−3^	5.82845 × 10^−2^
2500	1.0014 × 10^−2^	9.6775 × 10^−3^	0.4440	2.17211	9.58249 × 10^−3^	5.92091 × 10^−2^
5000	1.0154 × 10^−2^	9.4905 × 10^−3^	4.997 × 10^−2^	2.08184	9.14869 × 10^−3^	5.90042 × 10^−2^
7500	1.0025 × 10^−2^	9.4971 × 10^−3^	1.205 × 10^−2^	2.05869	9.03097 × 10^−3^	5.77781 × 10^−2^
10,000	1.0055 × 10^−2^	9.5011 × 10^−3^	1.524 × 10^−2^	2.06152	8.9892 × 10^−3^	5.73231 × 10^−2^
20,000	8.9041 × 10^−3^	9.5659 × 10^−3^	3.013 × 10^−2^	2.00109	8.52489 × 10^−3^	5.42623 × 10^−2^
50,000	9.2309 × 10^−3^	9.5375 × 10^−3^	5.459 × 10^−3^	2.02138	8.63519 × 10^−3^	5.50748 × 10^−2^
100,000	9.6972 × 10^−3^	9.5093 × 10^−3^	3.493 × 10^−3^	2.04844	8.83866 × 10^−3^	5.64516 × 10^−2^

**Table 2:** The elements $E_{1N}^{\hat{\lambda }}$, $v_{1N}^{\hat{\lambda }}$, $E_{2N}^{\hat{\lambda }}$, $v_{2N}^{\hat{\lambda }}$ and $E_{\infty N}^{\hat{\lambda }}$ of the vector $g_{N}^{\hat{\lambda }}$ solving optimization task (13) for regularization parameter $\hat{\lambda }=2.51\times 10^{-3}$, the mean-square identification indices $Q_{N}\left(g_{N}^{\hat{\lambda }}\right)$, Equation (8), the integral quadratic indices $Q\left(g_{N}^{\hat{\lambda }}\right)$, Equation (5), and the relative square errors ERR (39) of the measurement-independent parameters $g^{*\lambda }$, approximation for five-parameter Maxwell model (26) and N relaxation modulus measurements corrupted by additive independent noises of normal distribution with standard deviation σ=0.001 [Pa].

N	$Q_{N}\left(g_{N}^{\hat{\lambda }}\right)\;\left[Pa^{2}\right]$	$Q\left(g_{N}^{\hat{\lambda }}\right)\;\left[Pa^{2}\right]$	ERR [%]	$E_{1N}^{\hat{\lambda }}\;\left[Pa\right]$	$v_{1N}^{\hat{\lambda }}\;\left[s^{-1}\right]$	$E_{2N}^{\hat{\lambda }}\;\left[Pa\right]$	$v_{2N}^{\hat{\lambda }}\;\left[s^{-1}\right]$	$E_{\infty N}^{\hat{\lambda }}\;\left[Pa\right]$
50	5.0 × 10^−6^	6.8155 × 10^−4^	2.07	0.94119	3.57829 × 10^−3^	1.73538	3.09241 × 10^−2^	8.412 × 10^−3^
100	3.6965 × 10^−5^	5.4636 × 10^−5^	1.75 × 10^−2^	1.01745	3.85167 × 10^−3^	2.02555	4.15854 × 10^−2^	1.2639 × 10^−2^
250	4.5229 × 10^−5^	5.5365 × 10^−5^	1.06 × 10^−2^	1.01928	3.85649 × 10^−3^	2.07666	4.22544 × 10^−2^	1.2885 × 10^−2^
500	5.1215 × 10^−5^	5.0257 × 10^−5^	3.05 × 10^−3^	1.02616	3.89451 × 10^−3^	2.06699	4.28871 × 10^−2^	1.2885 × 10^−2^
1000	5.1036 × 10^−5^	5.0021 × 10^−5^	5.5 × 10^−3^	1.02949	3.88211 × 10^−3^	2.07118	4.33627 × 10^−2^	1.2897 × 10^−2^
2500	5.0049 × 10^−5^	4.9049 × 10^−5^	1.02 × 10^−3^	1.02709	3.87612 × 10^−3^	2.06167	4.30789 × 10^−2^	1.27968 × 10^−2^
5000	5.1942 × 10^−5^	4.8581 × 10^−5^	1.89 × 10^−4^	1.02982	3.88972 × 10^−3^	2.05571	4.31916 × 10^−2^	1.31245 × 10^−2^
7500	5.1596 × 10^−5^	4.8712 × 10^−5^	1.89 × 10^−4^	1.03037	3.89006 × 10^−3^	2.05869	4.32779 × 10^−2^	1.30726 × 10^−2^
10,000	5.1276 × 10^−5^	4.8781 × 10^−5^	5.62 × 10^−4^	1.02979	3.88757 × 10^−3^	2.05899	4.32133 × 10^−2^	1.29740 × 10^−2^
20,000	4.8846 × 10^−5^	4.8995 × 10^−5^	1.20 × 10^−4^	1.02589	3.87391 × 10^−3^	2.05207	4.29263 × 10^−2^	1.28128 × 10^−2^
50,000	4.9066 × 10^−5^	4.8934 × 10^−5^	7.72 × 10^−5^	1.02631	3.87501 × 10^−3^	2.05244	4.29290 × 10^−2^	1.27937 × 10^−2^
100,000	4.9971 × 10^−5^	4.8738 × 10^−5^	6.42 × 10^−6^	1.02757	3.88095 × 10^−3^	2.05430	4.29902 × 10^−2^	1.29231 × 10^−2^

**Table 3:** The elements $E_{1N}^{\hat{\lambda }}$, $v_{1N}^{\hat{\lambda }}$, $E_{2N}^{\hat{\lambda }}$, $v_{2N}^{\hat{\lambda }}$,$\;E_{3N}^{\hat{\lambda }}$, $v_{3N}^{\hat{\lambda }}$ and $E_{\infty N}^{\hat{\lambda }}$ of the vector $g_{N}^{\hat{\lambda }}$ solving optimization task (13) for regularization parameter $\hat{\lambda }=1.27\times 10^{-3}$, the mean-square identification indices $Q_{N}\left(g_{N}^{\hat{\lambda }}\right)$, Equation (8), the integral quadratic indices $Q\left(g_{N}^{\hat{\lambda }}\right)$, Equation (5), and the relative square errors ERR (39) of the measurement-independent parameters $g^{*\lambda }$, approximation for seven-parameter Maxwell model (26) and N relaxation modulus measurements corrupted by additive independent noises of normal distribution with standard deviation σ=0.001 [Pa].

N	$Q_{N}\left(g_{N}^{\hat{\lambda }}\right)\;\left[Pa^{2}\right]$	$Q\left(g_{N}^{\hat{\lambda }}\right)\;\left[Pa^{2}\right]$	ERR [%]	$E_{1N}^{\hat{\lambda }}\;\left[Pa\right]$	$v_{1N}^{\hat{\lambda }}\;\left[s^{-1}\right]$	$E_{2N}^{\hat{\lambda }}\;\left[Pa\right]$	$v_{2N}^{\hat{\lambda }}\;\left[s^{-1}\right]$	$E_{3N}^{\hat{\lambda }}\;\left[Pa\right]$	$v_{3N}^{\hat{\lambda }}\;\left[s^{-1}\right]$	$E_{\infty N}^{\hat{\lambda }}\;\left[Pa\right]$
50	1.6 × 10^−5^	1.08569 × 10^−3^	3.66 × 10^−2^	0.91380	3.496 × 10^−3^	0.83143	2.7811 × 10^−2^	0.82996	2.7811 × 10^−2^	7.603 × 10^−3^
100	2.25694 × 10^−5^	4.38001 × 10^−5^	7.83 × 10^−4^	0.96972	3.695 × 10^−3^	1.0064	2.832 × 10^−2^	1.05894	5.5844 × 10^−2^	1.0765 × 10^−2^
250	1.39206 × 10^−5^	1.13403 × 10^−5^	1.53 × 10^−5^	0.96278	3.675 × 10^−3^	1.04195	2.7757 × 10^−2^	1.10192	6.3315 × 10^−2^	1.0623 × 10^−2^
500	1.62056 × 10^−5^	1.96741 × 10^−5^	2.63 × 10^−5^	0.95898	3.665 × 10^−3^	1.03016	2.7044 × 10^−2^	1.09224	6.3563 × 10^−2^	1.045 × 10^−2^
1000	1.23718 × 10^−5^	9.7523 × 10^−6^	5.82 × 10^−5^	0.96012	3.664 × 10^−3^	1.04663	2.7517 × 10^−2^	1.10631	6.428 × 10^−2^	1.0313 × 10^−2^
2500	1.33721 × 10^−5^	1.09919 × 10^−5^	2.17 × 10^−5^	0.96250	3.67 × 10^−3^	1.04209	2.7724 × 10^−2^	1.10388	6.3365 × 10^−2^	1.0324 × 10^−2^
5000	1.36971 × 10^−5^	1.15176 × 10^−5^	1.82 × 10^−5^	0.96271	3.673 × 10^−3^	1.03686	2.7685 × 10^−2^	1.10569	6.3398 × 10^−2^	1.0428 × 10^−2^
7500	1.35309 × 10^−5^	1.12970 × 10^−5^	1.95 × 10^−5^	0.96231	3.67 × 10^−3^	1.03806	2.7636 × 10^−2^	1.10573	6.3676 × 10^−2^	1.0362 × 10^−2^
10,000	1.35952 × 10^−5^	1.14719 × 10^−5^	1.29 × 10^−5^	0.96236	3.67 × 10^−3^	1.04048	2.7686 × 10^−2^	1.10274	6.3591 × 10^−2^	1.033 × 10^−2^
20,000	1.58069 × 10^−5^	1.60772 × 10^−5^	1.19 × 10^−5^	0.963075	3.672 × 10^−3^	1.03044	2.7711 × 10^−2^	1.09572	6.2326 × 10^−2^	1.0375 × 10^−2^
50,000	1.53858 × 10^−5^	1.50425 × 10^−5^	2.82 × 10^−6^	0.96263	3.671 × 10^−3^	1.03389	2.7724 × 10^−2^	1.09609	6.2494 × 10^−2^	1.0336 × 10^−2^
100,000	1.45691 × 10^−5^	1.33615 × 10^−5^	2.62 × 10^−7^	0.96311	3.674 × 10^−3^	1.03655	2.7767 × 10^−2^	1.09878	6.2832 × 10^−2^	1.0407 × 10^−2^

**Table 4:** The elements $G_{0N}^{\hat{\lambda }}$, $v_{rN}^{\hat{\lambda }}$ and $\beta_{N}^{\hat{\lambda }}$ of the vector $g_{N}^{\hat{\lambda }}$ solving optimization task (13) for regularization parameter $\hat{\lambda }=1.31\times 10^{-3}$, the mean-square identification indices $Q_{N}\left(g_{N}^{\hat{\lambda }}\right)$, Equation (8), the integral quadratic indices $Q\left(g_{N}^{\hat{\lambda }}\right)$, Equation (5), and the relative square errors ERR (39) of the measurement-independent parameters $g^{*\lambda }$, approximation for KWW model (28) and N relaxation modulus measurements corrupted by additive independent noises of normal distribution with standard deviation σ=0.001 [Pa].

N	$Q_{N}\left(g_{N}^{\hat{\lambda }}\right)\;\left[Pa^{2}\right]$	$Q\left(g_{N}^{\hat{\lambda }}\right)\;\left[Pa^{2}\right]$	ERR [%]	$G_{0N}^{\hat{\lambda }}\;\left[Pa\right]$	$v_{rN}^{\hat{\lambda }}\;\left[s^{-1}\right]$	$\beta_{N}^{\hat{\lambda }}\;\left[-\right]$
50	3.60 × 10^−4^	7.8738 × 10^−4^	0.3578	3.72319	2.52024 × 10^−2^	0.45406
100	4.670 × 10^−4^	7.9693 × 10^−4^	1.1724	4.39118	3.72331 × 10^−2^	0.41084
250	5.1244 × 10^−4^	6.6349 × 10^−4^	8.07 × 10^−3^	3.99535	3.19162 × 10^−2^	0.42414
500	4.6738 × 10^−4^	7.8228 × 10^−4^	0.4555	3.69177	2.67951 × 10^−2^	0.4429
1000	5.7908 × 10^−4^	6.5920 × 10^−4^	0.017	3.90855	2.97799 × 10^−2^	0.43396
2500	6.5771 × 10^−4^	6.5127 × 10^−4^	2.44 × 10^−3^	3.98004	3.05131 × 10^−2^	0.43259
5000	7.4273 × 10^−4^	6.5982 × 10^−4^	2.919 × 10^−2^	3.89251	2.92203 × 10^−2^	0.43686
7500	7.3871 × 10^−4^	6.7181 × 10^−4^	8.346 × 10^−2^	3.84555	2.85530 × 10^−2^	0.43938
10,000	7.1466 × 10^−4^	6.6242 × 10^−4^	3.84 × 10^−2^	3.88251	2.91616 × 10^−2^	0.43676
20,000	6.2228 × 10^−4^	6.5372 × 10^−4^	5.98 × 10^−3^	3.92963	2.98969 × 10^−2^	0.43363
50,000	6.3356 × 10^−4^	6.4931 × 10^−4^	4.39 × 10^−4^	3.96871	3.05467 × 10^−2^	0.43129
100,000	6.4603 × 10^−4^	6.4783 × 10^−4^	4.67 × 10^−3^	3.98754	3.09393 × 10^−2^	0.42979

## Data Availability

Data are contained within the article.

## Appendix A

### Appendix A.1. Derivation of the Inequality (22)

Note that for any λ>0, by (14) and (15), we have

(A1) $$0\leq L\left(g_{N}^{\lambda },\lambda \right)-L\left(g^{*\lambda },\lambda \right)\leq L\left(g_{N}^{\lambda },\lambda \right)-L_{N}\left(g_{N}^{\lambda },\lambda \right)+L_{N}\left(g_{N}^{\lambda },\lambda \right)-L\left(g^{*\lambda },\lambda \right),$$

where the functional $L_{N}\left(g\right)$ is defined by Equation (10). Since, by (13) and (10), $L_{N}\left(g_{N}^{\lambda },\lambda \right)\leq L_{N}\left(g^{*\lambda },\lambda \right)$, inequality (A1) implies that

$$0\leq L\left(g_{N}^{\lambda },\lambda \right)-L\left(g^{*\lambda },\lambda \right)\leq L\left(g_{N}^{\lambda },\lambda \right)-L_{N}\left(g_{N}^{\lambda },\lambda \right)+L_{N}\left(g^{*\lambda },\lambda \right)-L\left(g^{*\lambda },\lambda \right),$$

which can be rewritten as

$$0\leq L\left(g_{N}^{\lambda },\lambda \right)-L\left(g^{*\lambda },\lambda \right)\leq L\left(g_{N}^{\lambda },\lambda \right)+\sigma^{2}-L_{N}\left(g_{N}^{\lambda },\lambda \right)+L_{N}\left(g^{*\lambda },\lambda \right)-\;L\left(g^{*\lambda },\lambda \right)-\sigma^{2},$$

whence the next estimation follows:

$$\left|L\left(g_{N}^{\lambda },\lambda \right)-L\left(g^{*\lambda },\lambda \right)\right|\leq 2\;\underset{g\in G}{\sup}\left|L\left(g,\lambda \right)+\sigma^{2}-L_{N}\left(g,\lambda \right)\right|.$$

The above, by (14) and (10), is equivalent to

(A2) $$\left|L\left(g_{N}^{\lambda },\lambda \right)-L\left(g^{*\lambda },\lambda \right)\right|\leq 2\underset{g\in G}{\sup}\left|Q\left(g\right)+\sigma^{2}-Q_{N}\left(g\right)\right|,$$

where the upper bound does not depend on the regularizer term R(g).

The relaxation modulus measurements are corrupted by noises $Z_{i}$ and ${\bar{G}}_{i}=G_{i}+Z_{i}$. Define for i=1,…,N i.i.d. random variables:

(A3) $$X_{i}\left(g\right)=Q\left(g\right)+\sigma^{2}-{\left[G_{i}+Z_{i}-G_{M}\left(T_{i},g\right)\right]}^{2}.$$

In view of Equation (7), $EX_{i}\left(g\right)=0$ for any g∈G. By Assumptions 1–4 and the fact that the weighting function ρ(t)≥0 is a density on T, there is a positive constant $\tilde{M}$ so that for any i=1,…,N and any g∈G, we have

(A4) $$\left|Q\left(g\right)-{\left[G_{i}-G_{M}\left(T_{i},g\right)\right]}^{2}\right|\leq \tilde{M},$$

whence, bearing in mind (A3), under Assumptions 5 and 6, for any i=1,…,N and g∈G, the next estimation follows:

(A5) $$\left|X_{i}\left(g\right)\right|\leq \tilde{M}+\sigma^{2}+\delta^{2}+2\theta \delta =\hat{M},$$

where the positive constant θ is such that $\left|G\left(T_{i}\right)-G_{M}\left(T_{i},g\right)\right|\leq \theta$ for every =1,…,N and g∈G. In view of Assumptions 1–4, such a constant exists, and it can be evaluated without difficulty: $\theta =M+M_{3}$ can be taken according to (4). Thus, random variables $X_{i}\left(g\right)$ are collectively bounded on the set G.

In view of (8) and (A3),

$$\left|Q\left(g\right)+\sigma^{2}-Q_{N}\left(g\right)\right|=\frac{1}{N}\left|\sum_{i=1}^{N}X_{i}\left(g\right)\right|.$$

Due to the compactness of G and the continuity of the indices Q(g) and $Q_{N}\left(g\right)$, there is a $\tilde{g}\in G$ so that

(A6) $$\underset{g\in G}{\sup}\left|Q\left(g\right)+\sigma^{2}-Q_{N}\left(g\right)\right|=\left|Q\left(\tilde{g}\right)+\sigma^{2}-Q_{N}\left(\tilde{g}\right)\right|=\frac{1}{N}\left|\sum_{i=1}^{N}X_{i}\left(\tilde{g}\right)\right|.$$

Now, combining (A2) and (A6), we have

(A7) $$\left|L\left(g_{N}^{\lambda },\lambda \right)-L\left(g^{*\lambda },\lambda \right)\right|\leq \frac{2}{N}\left|\sum_{i=1}^{N}X_{i}\left(\tilde{g}\right)\right|.$$

Since, keeping (A5) in mind, by using Hoeffding’s inequality [24], for every $\tilde{\varepsilon }>0$, we obtain

$$P\left\{\left|\sum_{i=1}^{N}X_{i}\left(\tilde{g}\right)\right|\geq \tilde{\varepsilon }\right\}\leq 2\exp\left(\frac{-2{\tilde{\varepsilon }}^{2}}{N{\left(2\hat{M}\right)}^{2}}\right),$$

and whence, by the inequality (A7), we finally have the following bound:

$$P\left\{\left|L\left(g_{N}^{\lambda },\lambda \right)-L\left(g^{*\lambda },\lambda \right)\right|\geq \varepsilon \right\}\leq P\left\{\left|\sum_{i=1}^{N}X_{i}\left(\tilde{g}\right)\right|\geq \frac{N\varepsilon }{2}\right\}\leq 2\exp\left(\frac{-N\varepsilon^{2}}{8{\hat{M}}^{2}}\right)=2\exp\left(\frac{-N\varepsilon^{2}}{8{\left(\tilde{M}+\sigma^{2}+\delta^{2}+2\theta \delta \right)}^{2}}\right)$$

for any ε>0, whence putting $\theta =M+M_{3}$, inequality (22) results with $\hat{M}$ (23) yielded by the right equality in (A5).

### Appendix A.2. Proof of Proposition 3

Let $g\neq g_{N}^{\hat{\lambda }}$ be an arbitrary admissible solution of the optimization task (25), whence $Q_{N}\left(g\right)\leq {\hat{Q}}_{N}$. Since $g_{N}^{\hat{\lambda }}$ solves (13) for the regularization parameter $\hat{\lambda }$, the following inequality holds:

$$Q_{N}\left(g_{N}^{\hat{\lambda }}\right)+\hat{\lambda }\;R\left(g_{N}^{\hat{\lambda }}\right)\leq Q_{N}\left(g\right)+\hat{\lambda }R\left(g\right),$$

which, bearing in mind that by (24), we have $Q_{N}\left(g_{N}^{\hat{\lambda }}\right)={\hat{Q}}_{N}$, yields

$${\hat{Q}}_{N}-Q_{N}\left(g\right)\leq \hat{\lambda }\left[R\left(g\right)-R\left(g_{N}^{\hat{\lambda }}\right)\right],$$

which together with the preceding implies that

$$R\left(g\right)\geq R\left(g_{N}^{\hat{\lambda }}\right),$$

for an arbitrary admissible g, i.e., $g_{N}^{\hat{\lambda }}$ is the solution of the optimization task (25).

### Appendix A.3. Proof of Lemma 1

By virtue of the identification index definition (8), the Schwarz inequality related to the scalar product of the vectors g and $Wg_{0}$ and the assumption $\left|G_{M}\left(t,g\right)\right|\leq p{\left\|g\right\|}_{2}$, the following inequality holds:

(A8) $$Q_{N}\left(g\right)+\lambda {\left(g-g_{0}\right)}^{T}W\left(g-g_{0}\right)\geq \frac{1}{N}\sum_{i=1}^{N}{\left[{\bar{G}}_{i}\right]}^{2}-\frac{2p}{N}\sum_{i=1}^{N}\left|{\bar{G}}_{i}\right|{\left\|g\right\|}_{2}+\lambda g^{T}Wg-\;2\lambda {\left\|g\right\|}_{2}{\left\|Wg_{0}\right\|}_{2}+\lambda g_{0}^{T}Wg_{0},$$

for any λ>0 and any $g\in {ℛ}_{+}^{K}$. Using the left of the known Rayleigh–Ritz inequalities [62] (Lemma I),

(A9) $$\lambda_{min}\left(X\right)x^{T}x\leq x^{T}Xx\leq \lambda_{max}\left(X\right)x^{T}x,$$

which holds for any $x\in {ℛ}^{K}$ and any symmetric matrix $X=X^{T}\in {ℛ}^{K,K}$, where $\lambda_{min}\left(X\right)$ and $\lambda_{max}\left(X\right)$ are minimal and maximal eigenvalues of X, and bearing in mind that in the case considered (Assumptions 1 and 6) of $\left|{\bar{G}}_{i}\right|\leq M+\delta$ for i=1,…,N, inequality (A8) yields

$$Q_{N}\left(g\right)+\lambda {\left(g-g_{0}\right)}^{T}W\left(g-g_{0}\right)\geq \frac{1}{N}\sum_{i=1}^{N}{\left[{\bar{G}}_{i}\right]}^{2}-\frac{2p}{N}\sum_{i=1}^{N}\left(M+\delta \right){\left\|g\right\|}_{2}+\;\lambda \;\lambda_{min}\left(W\right){\left\|g\right\|}_{2}^{2}-2\lambda {\left\|g\right\|}_{2}{\left\|Wg_{0}\right\|}_{2}+\lambda g_{0}^{T}Wg_{0}.$$

By Assumption 3, we have $G_{M}\left(t,0_{K}\right)=0$ for any t∈T, and therefore, Equation (8) immediately yields $Q_{N}\left(0_{K}\right)=\frac{1}{N}\sum_{i=1}^{N}{\left[{\bar{G}}_{i}\right]}^{2}$. Therefore, for any $g\in {ℛ}_{+}^{K}$ so that ${\left\|g\right\|}_{2}>\bar{M}$, where $\bar{M}$ is defined by (32), the inequality holds that

$$Q_{N}\left(g\right)+\lambda {\left(g-g_{0}\right)}^{T}W\left(g-g_{0}\right)>Q_{N}\left(0_{K}\right)+\lambda g_{0}^{T}Wg_{0},$$

whence the equivalence of the minimization tasks in Equation (33) follows. The proof is completed.

### Appendix A.4. Proof of Lemma 2

Via Equations (5) and (11), bearing in mind the left hand side inequality (A9), we have

(A10) $$Q\left(g\right)+\lambda {\left(g-g_{0}\right)}^{T}W\left(g-g_{0}\right)\geq \int_{T}G{\left(t\right)}^{2}\rho \left(t\right)dt-2\int_{T}G\left(t\right)G_{M}\left(t,g\right)\rho \left(t\right)dt+\;\lambda \;\lambda_{min}\left(W\right){\left\|g\right\|}_{2}^{2}-2\lambda {\left\|g\right\|}_{2}{\left\|Wg_{0}\right\|}_{2}+\lambda g_{0}^{T}Wg_{0}.$$

Via Assumption 1 and due to the non-negative definiteness of the true relaxation modulus and the relaxation modulus model, the following inequality holds:

$$\int_{T}G\left(t\right)G_{M}\left(t,g\right)\rho \left(t\right)dt\leq M\int_{T}G_{M}\left(t,g\right)\rho \left(t\right)dt,$$

which in view of the assumption $\left|G_{M}\left(t,g\right)\right|\leq p{\left\|g\right\|}_{2}$ and the equality $\int_{T}\rho \left(t\right)dt=1$ yield

$$\int_{T}G\left(t\right)G_{M}\left(t,g\right)\rho \left(t\right)dt\leq Mp{\left\|g\right\|}_{2}.$$

Therefore, by inequality (A10), we have

$$Q\left(g\right)+\lambda R\left(g\right)\geq \int_{T}G{\left(t\right)}^{2}\rho \left(t\right)dt-2Mp{\left\|g\right\|}_{2}+\lambda \;\lambda_{min}\left(W\right){\left\|g\right\|}_{2}^{2}-\;2\lambda {\left\|g\right\|}_{2}{\left\|Wg_{0}\right\|}_{2}+\lambda g_{0}^{T}Wg_{0}.$$

Since $R\left(0_{K}\right)=g_{0}^{T}Wg_{0}$, the above inequality means that if ${\left\|g\right\|}_{2}>\bar{\bar{M}}$, where $\bar{\bar{M}}$ is given by (35), then $Q\left(g\right)+\lambda R\left(g\right)>Q\left(0_{K}\right)+\lambda R\left(0_{K}\right)$, i.e., the equivalence of two optimization tasks in Equation (36) is now proved.

## Appendix B

### Appendix B.1. Data Concerning the GMA Rule and Optimal Model Parameter $g^{*\hat{\lambda }}$ for the Three-Parameter Maxwell Model (26)

**Table A1:** The elements ${\bar{E}}_{1N}$, ${\bar{v}}_{1N}$ and ${\bar{E}}_{\infty N}$ of the reference parameter vector ${\bar{g}}_{N}$ of three-parameter Maxwell model (26), minimizing (for N=50) the mean quadratic identification index $Q_{N}\left(g_{N}\right)$ (8), the minimal index $Q_{N}\left({\bar{g}}_{N}\right)$ and the model approximation index ${\hat{Q}}_{N}$ that are assumed for GMA rule (24), optimal regularization parameters $\hat{\lambda }$ for the standard deviations σ=0.001, 0.004, 0.007 [Pa], parameters $E_{1}^{*\hat{\lambda }}$, $v_{1}^{*\hat{\lambda }}$ and $E_{\infty }^{*\hat{\lambda }}$ of the optimal regularized models solving optimization task (15) for respective regularization parameters $\hat{\lambda }$, integral model approximation indices $Q\left(g^{*\hat{\lambda }}\right)$, Equation (5). In the last raw diagonal elements $w_{kk}$, k=1,2,3 of the weight matrix W (38) are given.

σ [Pa]	$\hat{\lambda }$	$Q_{N}\left({\bar{g}}_{N}\right)\;\left[Pa^{2}\right]$	${\hat{Q}}_{N}\;\left[Pa^{2}\right]$	${\bar{E}}_{1N}\;\left[Pa\right]$	${\bar{v}}_{1N}\;\left[s^{-1}\right]$	${\bar{E}}_{\infty N}\;\left[Pa\right]$
0.001	1.568 × 10^−2^	4.1107 × 10^−3^	0.0042	1.85421	7.21454 × 10^−3^	5.6666 × 10^−2^
0.004	1.72 × 10^−2^	4.2363 × 10^−3^	0.0043	1.85204	7.18845 × 10^−3^	5.70563 × 10^−2^
0.007	1.79 × 10^−2^	4.3894 × 10^−3^	0.0045	1.84983	7.16194 × 10^−3^	5.7437 × 10^−2^
σ [Pa]	$\hat{\lambda }$	$Q\left(g^{*\hat{\lambda }}\right)\;\left[Pa^{2}\right]$		$E_{1}^{*\hat{\lambda }}\;\left[Pa\right]$	$v_{1}^{*\hat{\lambda }}\;\left[s^{-1}\right]$	$E_{\infty }^{*\hat{\lambda }}\;\left[Pa\right]$
0.001	1.568 × 10^−2^	9.5165 × 10^−3^		2.03641	8.77502 × 10^−3^	5.5975 × 10^−2^
0.004	1.72 × 10^−2^	9.5368 × 10^−3^		2.03459	8.74101 × 10^−3^	5.48663 × 10^−2^
0.007	1.79 × 10^−2^	9.5464 × 10^−3^		2.03377	8.72578 × 10^−3^	5.43696 × 10^−2^
				$w_{11}$	$w_{22}$	$w_{33}$
				0.001	1	10

### Appendix B.2. Data Concerning the GMA Rule and Optimal Model Parameter $g^{*\hat{\lambda }}$ for the Five-Parameter Maxwell Model (26)

**Table A2:** The elements ${\bar{E}}_{1N}$, ${\bar{v}}_{1N}$, ${\bar{E}}_{2N},$ ${\bar{v}}_{2N}$ and ${\bar{E}}_{\infty N}$ of the reference vector ${\bar{g}}_{N}$ for five-parameter Maxwell model (26), minimizing for N=50 the identification index $Q_{N}\left(g_{N}\right)$ (8), the minimal index $Q_{N}\left({\bar{g}}_{N}\right)$, model approximation index ${\hat{Q}}_{N}$ assumed for GMA rule (24), optimal regularization parameters $\hat{\lambda }$ for the standard deviations σ=0.001, 0.004, 0.007 [Pa], parameters $E_{1}^{*\hat{\lambda }}$, $v_{1}^{*\hat{\lambda }}$, $E_{2}^{*\hat{\lambda }}$, $v_{2}^{*\hat{\lambda }}$ and $E_{\infty }^{*\hat{\lambda }}$ of the optimal regularized models solving optimization task (15) for respective regularization parameters $\hat{\lambda }$, integral model approximation indices $Q\left(g^{*\hat{\lambda }}\right)$, Equation (5). In the last raw diagonal elements $w_{kk}$, k=1,…, 5 of the weight matrix W (38) are given.

σ [Pa]	$\hat{\lambda }$	$Q_{N}\left({\bar{g}}_{N}\right)\;\left[Pa^{2}\right]$	${\hat{Q}}_{N}\;\left[Pa^{2}\right]$	${\bar{E}}_{1N}\;\left[Pa\right]$	${\bar{v}}_{1N}\;\left[s^{-1}\right]$	${\bar{E}}_{2N}\;\left[Pa\right]$	${\bar{v}}_{2N}\;\left[s^{-1}\right]$	${\bar{E}}_{\infty N}\;\left[Pa\right]$
0.001	2.51 × 10^−3^	4.4403 × 10^−6^	5.0 × 10^−6^	0.95212	3.63025 × 10^−3^	1.74059	3.16799 × 10^−2^	9.84 × 10^−3^
0.004	2.62 × 10^−3^	1.9897 × 10^−6^	2.0 × 10^−5^	0.93879	3.55242 × 10^−3^	1.73906	3.08364 × 10^−2^	8.473 × 10^−3^
0.007	2.77 × 10^−3^	6.0773 × 10^−5^	6.12 × 10^−5^	0.92754	3.48427 × 10^−3^	1.73970	3.01797 × 10^−2^	7.322 × 10^−3^
σ [Pa]	$\hat{\lambda }$	$Q\left(g^{*\hat{\lambda }}\right)\;\left[Pa^{2}\right]$		$E_{1}^{*\hat{\lambda }}\;\left[Pa\right]$	$v_{1}^{*\hat{\lambda }}\;\left[s^{-1}\right]$	$E_{2}^{*\hat{\lambda }}\;\left[Pa\right]$	$v_{2}^{*\hat{\lambda }}\;\left[s^{-1}\right]$	$E_{\infty }^{*\hat{\lambda }}\;\left[Pa\right]$
0.001	2.51 × 10^−3^	4.8799 × 10^−5^		1.02699	3.87801 × 10^−3^	2.05434	4.29624 × 10^−2^	1.287 × 10^−2^
0.004	2.62 × 10^−3^	4.8841 × 10^−5^		1.02679	3.87669 × 10^−3^	2.05449	4.29512 × 10^−2^	1.2824 × 10^−2^
0.007	2.77 × 10^−3^	4.8851 × 10^−5^		1.02709	3.87711 × 10^−3^	2.05427	4.29646 × 10^−2^	1.2787 × 10^−2^
				$w_{11}$	$w_{22}$	$w_{33}$	$w_{44}$	$w_{55}$
				0.01	1	0.0001	0.1	10

### Appendix B.3. Data Concerning the GMA Rule and Optimal Model Parameter $g^{*\hat{\lambda }}$ for the Seven-Parameter Maxwell Model (26)

**Table A3:** The elements ${\bar{E}}_{1N}$, ${\bar{v}}_{1N}$, ${\bar{E}}_{2N},$ ${\bar{v}}_{2N}$, ${\bar{E}}_{3N}$, ${\bar{v}}_{3N}$ and ${\bar{E}}_{\infty N}$ of the reference vector ${\bar{g}}_{N}$ of seven-parameter Maxwell model (26), minimizing (for N=50) identification index $Q_{N}\left(g_{N}\right)$ (8), the minimal index $Q_{N}\left({\bar{g}}_{N}\right)$ and model approximation index ${\hat{Q}}_{N}$ assumed for GMA rule (24), optimal regularization parameters $\hat{\lambda }$ for the standard deviations σ=0.001, 0.004, 0.007 [Pa], parameters $E_{1}^{*\hat{\lambda }}$, $v_{1}^{*\hat{\lambda }}$, $E_{2}^{*\hat{\lambda }}$, $v_{2}^{*\hat{\lambda }}$, $E_{3}^{*\hat{\lambda }}$, $v_{3}^{*\hat{\lambda }}$ and $E_{\infty }^{*\hat{\lambda }}$ of the optimal regularized models solving optimization task (15) for respective parameters $\hat{\lambda }$, integral model approximation indices $Q\left(g^{*\hat{\lambda }}\right)$, Equation (5). In the last raw diagonal elements $w_{kk}$, k=1,…, 7 of the weight matrix W (38) are given.

σ [Pa]	$\hat{\lambda }$	$Q_{N}\left({\bar{g}}_{N}\right)\;\left[Pa^{2}\right]$	${\hat{Q}}_{N}\;\left[Pa^{2}\right]$	${\bar{E}}_{1N}\;\left[Pa\right]$	${\bar{v}}_{1N}\;\left[s^{-1}\right]$	${\bar{E}}_{2N}\;\left[Pa\right]$	${\bar{v}}_{2N}\;\left[s^{-1}\right]$	${\bar{E}}_{3N}\;\left[Pa\right]$	${\bar{v}}_{3N}\;\left[s^{-1}\right]$	${\bar{E}}_{\infty N}\;\left[Pa\right]$
0.001	1.27 × 10^−3^	2.1028 × 10^−6^	1.6 × 10^−5^	0.9191	3.532 × 10^−3^	0.72158	2.0778 × 10^−2^	1.42317	5.5709 × 10^−2^	8.427 × 10^−3^
0.004	9.2 × 10^−4^	1.9802 × 10^−5^	2.6 × 10^−5^	0.93211	3.533 × 10^−3^	0.89967	2.5898 × 10^−2^	0.9034	3.9878 × 10^−2^	8.178 × 10^−3^
0.007	1.02 × 10^−3^	6.0766 × 10^−5^	6.745 × 10^−5^	0.92678	3.479 × 10^−3^	0.90175	3.0131 × 10^−2^	0.8378	3.0130 × 10^−2^	7.115 × 10^−3^
σ [Pa]	$\hat{\lambda }$	$Q\left(g^{*\hat{\lambda }}\right)\;\left[Pa^{2}\right]$		$E_{1}^{*\hat{\lambda }}\;\left[Pa\right]$	$v_{1}^{*\hat{\lambda }}\;\left[s^{-1}\right]$	$E_{2}^{*\hat{\lambda }}\;\left[Pa\right]$	$v_{2}^{*\hat{\lambda }}\;\left[s^{-1}\right]$	$E_{3}^{*\hat{\lambda }}\;\left[Pa\right]$	$v_{3}^{*\hat{\lambda }}\;\left[s^{-1}\right]$	$E_{\infty }^{*\hat{\lambda }}\;\left[Pa\right]$
0.001	1.27 × 10^−3^	1.3786 × 10^−5^		0.96267	3.671 × 10^−3^	1.03609	2.7733 × 10^−2^	1.09812	6.2789 × 10^−2^	1.0358 × 10^−2^
0.004	9.2 × 10^−4^	9.2425 × 10^−6^		0.95907	3.659 × 10^−3^	1.11782	6.445 × 10^−2^	1.03771	2.7318 × 10^−2^	1.0186 × 10^−2^
0.007	1.02 × 10^−3^	1.1645 × 10^−5^		0.96314	3.673 × 10^−3^	1.03848	2.7766 × 10^−2^	1.10342	6.3286 × 10^−2^	1.0399 × 10^−2^
				$w_{11}$	$w_{22}$	$w_{33}$	$w_{44}$	$w_{55}$	$w_{66}$	$w_{77}$
				0.1	10	0.1	1	0.1	1	10

### Appendix B.4. Data Concerning the GMA Rule and Optimal Model Parameter $g^{*\hat{\lambda }}$ for the KWW Model (28)

**Table A4:** The elements ${\bar{G}}_{0N}$, ${\bar{v}}_{rN}$ and ${\bar{\beta }}_{N}$ of the reference vector ${\bar{g}}_{N}$ of the KWW model (28), minimizing for N=50 identification index $Q_{N}\left(g_{N}\right)$ (8), the index $Q_{N}\left({\bar{g}}_{N}\right)$ and the model approximation index ${\hat{Q}}_{N}$ assumed for GMA rule (24), optimal regularization parameters $\hat{\lambda }$ for the standard deviations σ=0.001, 0.004, 0.007 [Pa], parameters $G_{0}^{*\hat{\lambda }}$, $v_{r}^{*\hat{\lambda }}$ and $\beta^{*\hat{\lambda }}$ of the optimal regularized models solving optimization task (15) for respective $\hat{\lambda }$, integral model approximation indices $Q\left(g^{*\hat{\lambda }}\right)$ (5). In the last raw diagonal elements $w_{kk}$, k=1,2,3 of the weight matrix W (38) are given.

σ [Pa]	$\hat{\lambda }$	$Q_{N}\left({\bar{g}}_{N}\right)\;\left[Pa^{2}\right]$	${\hat{Q}}_{N}\;\left[Pa^{2}\right]$	${\bar{G}}_{0N}\;\left[Pa\right]$	${\bar{v}}_{rN}\;\left[s^{-1}\right]$	${\bar{\beta }}_{N}\;\left[-\right]$
0.001	1.31 × 10^−3^	3.4881 × 10^−4^	3.60 × 10^−4^	3.99652	2.96219 × 10^−2^	0.43412
0.004	3.01 × 10^−3^	3.8915 × 10^−4^	3.91 × 10^−4^	4.01451	2.98816 × 10^−2^	0.43236
0.007	2.87 × 10^−3^	4.5689 × 10^−4^	4.96 × 10^−4^	4.03218	3.01369 × 10^−2^	0.43064
σ [Pa]	$\hat{\lambda }$	$Q\left(g^{*\hat{\lambda }}\right)\;\left[Pa^{2}\right]$		$G_{0}^{*\hat{\lambda }}\;\left[Pa\right]$	$v_{r}^{*\hat{\lambda }}\;\left[s^{-1}\right]$	$\beta^{*\hat{\lambda }}\;\left[-\right]$
0.001	1.31 × 10^−3^	6.4994 × 10^−4^		3.96037	3.04633 × 10^−2^	0.43159
0.004	3.01 × 10^−3^	6.6262 × 10^−4^		3.87901	2.89852 × 10^−2^	0.43771
0.007	2.87 × 10^−3^	6.6129 × 10^−4^		3.88528	2.90969 × 10^−2^	0.43723
				$w_{11}$	$w_{22}$	$w_{33}$
				0.02	1	0.1

## References

[1] Ferry J.D. (1980). Viscoelastic Properties of Polymers.

[2] Malkin A.I.A., Malkin A.Y., Isayev A.I. (2006). Rheology: Concepts, Methods and Applications.

[3] Duan X., Yuan H., Tang W., He J., Guan X. (2022). An Engineering Prediction Model for Stress Relaxation of Polymer Composites at Multiple Temperatures. Polymers.

[4] Lei D., Liang Y., Xiao R. (2018). A fractional model with parallel fractional Maxwell elements for amorphous thermoplastics. Phys. A Stat. Mech. Its Appl..

[5] Fancey K.S. (2005). A mechanical model for creep, recovery and stress relaxation in polymeric materials. J. Mater. Sci..

[6] Tang D., Marchesini F.H., Cardon L., D’hooge D.R. (2020). State of the-Art for Extrudate Swell of Molten Polymers: From Fundamental Understanding at Molecular Scale toward Optimal Die Design at Final Product Scale. Macromol. Mater. Eng..

[7] Dealy J.M., Read D.J., Larson R.G. (2018). Structure and Rheology of Molten Polymers.

[8] Ishii A. (2021). Spatial and temporal heterogeneity of Kohlrausch–Williams–Watts stress relaxations in metallic glasses. Comput. Mater. Sci..

[9] Lukichev A. (2019). Physical meaning of the stretched exponential Kohlrausch function. Phys. Lett. A.

[10] Ljung L. (1999). System Identification: Theory for the User.

[11] Tikhonov A.N., Arsenin V.Y. (1977). Solutions of Ill-posed Problems.

[12] Hansen P.C. (1998). Rank-Deficient and Discrete Ill-Posed Problems.

[13] Ljung L. (1978). Convergence analysis of parametric identification methods. IEEE Trans. Autom. Control.

[14] Hasiewicz Z., Stankiewicz A. (1985). On input-dependent system identification by Monte Carlo approach. IEEE Trans. Autom. Control.

[15] Kwakye-Nimo S., Inn Y., Yu Y., Wood-Adams P.M. (2022). Linear viscoelastic behavior of bimodal polyethylene. Rheol. Acta.

[16] Muzeau E., Perez J., Johari G.P. (1991). Mechanical spectrometry of the beta-relaxation in poly(methyl methacrylate). Macromolecules.

[17] Pérez-Calixto D., Amat-Shapiro S., Zamarrón-Hernández D., Vázquez-Victorio G., Puech P.-H., Hautefeuille M. (2021). Determination by Relaxation Tests of the Mechanical Properties of Soft Polyacrylamide Gels Made for Mechanobiology Studies. Polymers.

[18] Lorenzo G., Checmarev G., Zaritzky N., Califano A. (2011). Linear viscoelastic assessment of cold gel-like emulsions stabilized with bovine gelatin. LWT—Food Sci. Technol..

[19] Cirillo G., Spizzirri U.G., Iemma F. (2015). Functional Polymers in Food Science: From Technology to Biology, Volume 1: Food Packaging.

[20] Carrillo-Navas H., Hernández-Jaimes C., Utrilla-Coello R.G., Meraz M., Vernon-Carter E.J., Alvarez-Ramirez J. (2014). Viscoelastic relaxation spectra of some native starch gels. Food Hydrocoll..

[21] Andrei N. (2022). Modern Numerical Nonlinear Optimization.

[22] Kaltenbacher B., Neubauer A., Scherzer O. (2008). Iterative Regularization Methods for Nonlinear Ill-Posed Problems.

[23] Hofmann B., Hofmann C., Mathé P., Plato R., Baasansuren J., Khan A.A., Migórski S.S., Sama M. (2021). Nonlinear Tikhonov Regularization in Hilbert Scales with Oversmoothing Penalty: Inspecting Balancing Principles. Deterministic and Stochastic Optimal Control and Inverse Problems.

[24] Hoeffding W. (1963). Probability inequalities for sums of bounded random variables. J. Am. Stat. Assoc..

[25] Chavent G., Kunisch K. (1994). Convergence of Tikhonov regularization for constrained ill-posed inverse problems. Inverse Probl..

[26] Chavent G., Kunisch K. (1998). State-Space Regularization: Geometric Theory. Appl. Math. Optim..

[27] Pillonetto G., Chen T., Chiuso A., De Nicolao G., Ljung L. (2022). Regularized System Identification: Learning Dynamic Models from Data.

[28] Stankiewicz A. (2003). A scheme for identification of continuous relaxation time spectrum of biological viscoelastic materials. Acta Sci. Pol. Ser. Tech. Agrar..

[29] Stankiewicz A. (2010). How to mollify the ill posedness of the problem of Maxwell model identification of viscoelastic plant materials. Acta Agrophysica.

[30] Lee S., Knauss W. (2000). A Note on the Determination of Relaxation and Creep Data from Ramp Tests. Mech. Time-Depend. Mater..

[31] Tscharnuter D., Jerabek M., Major Z., Lang R.W. (2011). On the determination of the relaxation modulus of PP compounds from arbitrary strain histories. Mech. Time-Depend. Mater..

[32] Sorvari J., Malinen M. (2006). Determination of the relaxation modulus of a linearly viscoelastic material. Mech. Time-Depend. Mater..

[33] Zapas L.J., Craft T. (1965). Correlation of Large Longitudinal Deformations With Different Strain Histories. J. Res. Natl. Bur. Stand. A Phys Chem..

[34] Martynova E.D., Stetsenko N.S. (2018). Identification of Behavior of Linearly Viscoelastic Materials from Experiments on Relaxation with Initial Area of Increasing Strain. J. Mach. Manuf. Reliab..

[35] Brazel C.S., Rosen S.L. (2012). Fundamental Principles of Polymeric Materials.

[36] Kapnistos M., Lang M., Vlassopoulos D., Pyckhout-Hintzen W., Richter D., Cho D., Chang T., Rubinstein M. (2008). Unexpected power-law stress relaxation of entangled ring polymers. Nat. Mater..

[37] Bonfanti A., Kaplan J.L., Charras G., Kabla A. (2020). Fractional viscoelastic models for power-law materials. Soft Matter.

[38] Mainardi F. (2010). Fractional Calculus and Waves in Linear Viscoelasticity: An Introduction to Mathematical Models.

[39] Schippers C., Tsarkova L.A., Bahners T., Gutmann J.S., Cleve E. (2021). Improved Maxwell Model Approach and its Applicability toward Lifetime Prediction of Biobased Viscoelastic Fibers. Macromol. Mater. Eng..

[40] Wen L., Wang j., Guo Y., Hu W. (2021). Role of stress relaxation in stress-induced polymer crystallization. Polymer.

[41] Commins T., Siviour C.R. (2023). Stress relaxation after low-and high-rate deformation of polyurethanes. Proc. R. Soc. A.

[42] Bihari B.K., Kumaraswamy A., Jain M., Kurva R., Vipin L. (2023). Simulation of Stress Relaxation Behaviour of Composite Propellants with Varying Solid Loading Using the Generalized Maxwell Model. Cent. Eur. J. Energ. Mater..

[43] Cholleti E.R., Stringer J., Kelly P., Bowen C., Aw K. (2020). The effect of barium titanate ceramic loading on the stress relaxation behavior of barium titanate-silicone elastomer composites. Polym. Eng. Sci..

[44] Bochnia J., Kozior T., Szot W., Rudnik M., Zmarzły P., Gogolewski D., Szczygieł P., Musiałek M. (2022). Selected Mechanical and Rheological Properties of Medical Resin MED610 in PolyJet Matrix Three-Dimensional Printing Technology in Quality Aspects. 3D Print. Addit. Manuf..

[45] Feng C., Zheng J., Wang Y., Zhang C., Ming P. (2023). Viscosity mechanism of perfluorosulfonic acid-based materials and their application in proton exchange membrane fuel cells. Appl. Mater. Today.

[46] Salimi A., Abbassi-Sourki F., Karrabi M., Ghoreishy M.H.R. (2021). Investigation on viscoelastic behavior of virgin EPDM/reclaimed rubber blends using Generalized Maxwell Model (GMM). Polym. Test..

[47] Alvarez F., Alegra A., Colmenero J. (1991). Relationship between the time-domain Kohlrausch-Williams-Watts and frequency-domain Havriliak-Negami relaxation functions. Phys. Rev. B.

[48] Aydiner E. (2019). A Simple Model for Stretched Exponential Relaxation in Three-Level Jumping Process. Phys. Status Solidi B.

[49] Neuber N., Gross O., Frey M., Bochtler B., Kuball A., Hechler S., Yang F., Pineda E., Westermeier F., Sprung M. (2022). Disentangling structural and kinetic components of the α-relaxation in supercooled metallic liquids. Commun. Phys..

[50] Jeong D., Yook J., Kwon D.-S., Shim J., Lee J.-C. (2023). Interweaving Elastic and Hydrogen Bond-Forming Polymers into Highly Tough and Stress-Relaxable Binders for High-Performance Silicon Anode in Lithium-Ion Batteries. Adv. Sci..

[51] Singh O., Venugopal P.P., Chakraborty D. (2023). Effect of Water Models on The Stability of RNA: Role of Counter-Ions. Chem. Phys. Impact.

[52] Minecka A., Tarnacka M., Jurkiewicz K., Żakowiecki D., Kamiński K., Kamińska E. (2023). Mesoporous Matrices as a Promising New Generation of Carriers for Multipolymorphic Active Pharmaceutical Ingredient Aripiprazole. Mol. Pharm..

[53] Saranya P., Vanitha D., Sundaramahalingam K., Nandhinilakshmi M., Samad S.A. (2023). Structural and electrical properties of cross-linked blends of Xanthan gum and polyvinylpyrrolidone-based solid polymer electrolyte. Ionics.

[54] Tong Y., Li F., Song L., Liu Y., Huo J., Qiao J., Yao Y., Pelletier J.M., Crespo D., Pineda E. (2024). Unexpected non-monotonic changing in the heterogeneity of glasses during annealing. J. Mater. Sci. Technol..

[55] Maurer J., Jerabek M., Salaberger D., Thor M., Kastner J., Major Z. (2022). Stress relaxation behaviour of glass fibre reinforced thermoplastic composites and its application to the design of interrupted in situ tensile tests for investigations by X-ray computed tomography. Polym. Test..

[56] Golub G.H., Van Loan C.F. (1996). Matrix Computations.

[57] Wang J., Wang X., Ruan H. (2020). On the mechanical β relaxation in glass and its relation to the double-peak phenomenon in impulse excited vibration at high temperatures. J. Non-Cryst. Solids.

[58] Bardet S., Gril J. (2002). Modelling the transverse viscoelasticity of green wood using a combination of two parabolic elements. C. R. Mécanique.

[59] Kurenuma Y., Nakano T. (2012). Analysis of stress relaxation on the basis of isolated relaxation spectrum for wet wood. J. Mater. Sci..

[60] Stankiewicz A. (2023). Two-Level Scheme for Identification of the Relaxation Time Spectrum Using Stress Relaxation Test Data with the Optimal Choice of the Time-Scale Factor. Materials.

[61] Stankiewicz A., Bojanowska M., Drozd P. (2023). On Recovery of a Non-Negative Relaxation Spectrum Model from the Stress Relaxation Test Data. Polymers.

[62] Lee C.-H. (1996). Upper and lower matrix bounds of the solution for the discrete Lyapunov equation. IEEE Trans. Autom. Control..

